# Macronutrient Intake and Clinical Manifestations in Adults with Long COVID-19: A Cross-Sectional Analysis of the BioICOPER Study

**DOI:** 10.3390/nu18213716

**Published:** 2026-11-09

**Authors:** David Rodríguez-García, Marta Gómez-Sánchez, Elena Navarro-Matías, Silvia Arroyo-Romero, Alicia Navarro-Cáceres, Nuria Suárez-Moreno, Andrea Domínguez-Martín, Manuel A. Gómez-Marcos, David Arjol, Leticia Gómez-Sánchez, BioICOPER Investigators Group

**Affiliations:** 1 Primary Care Research Unit of Salamanca (APISAL), Salamanca Primary Care Management, Regional Health Authority of Castilla y León (SACyL), Avenida de Portugal 83, 37005 Salamanca, Spain; david8rg@usal.es (D.R.-G.); enavarro@saludcastillayleon.es (E.N.-M.); silvia_ar@usal.es (S.A.-R.); alicia.nav@usal.es (A.N.-C.); nuria.suarez@usal.es (N.S.-M.); andreadm@usal.es (A.D.-M.); 2 Institute of Biomedical Research of Salamanca (IBSAL), Paseo de San Vicente, 58-182, 37007 Salamanca, Spain; 3 Department of Medicine, University of Salamanca, Calle Alfonso X el Sabio s/n, 37007 Salamanca, Spain; 4 Home Hospitalization Service, Hospital Universitario Marqués de Valdecilla, 39008 Santander, Spain; martagmzsnchz@gmail.com; 5 Research Network on Chronicity, Primary Care and Health Promotion (RICAPPS), Avenida de Portugal 83, 37005 Salamanca, Spain; 6 Emergency Department, Hospital Universitario Príncipe de Asturias Alcalá, Carretera de Meco, s/n ( University of Alcalá Outer Campus), 28805 Madrid, Spain

**Keywords:** long COVID-19, macronutrients, dietary intake, frailty, cognitive function

## Abstract

**Background/Objectives**: The relationship between macronutrient intake and the clinical manifestations of long COVID-19 remains poorly understood. This study examined these associations in adults from the BioICOPER cohort. **Methods**: This cross-sectional study included 290 adults with long COVID-19 from the BioICOPER cohort who had complete seven-day dietary records collected using the EVIDENT application. Twelve clinical outcomes were assessed using linear regression with robust standard errors, ordinal logistic regression for frailty, and multinomial logistic regression for dyspnea. Model 1 examined eight exposures (total energy and seven nutrient intake variables), adjusting for age and sex. Model 2 examined seven exposures expressed as energy percentages or fiber density, additionally adjusting for total energy intake. Benjamini–Hochberg correction was applied separately to the 96 and 84 tests, respectively. **Results**: In Model 1, higher intakes of total fat (odds ratio [OR]: 0.983; 95% confidence interval [CI]: 0.974–0.992), saturated fatty acids (OR: 0.956; 95% CI: 0.932–0.981), and monounsaturated fatty acids (OR: 0.967; 95% CI: 0.948–0.987), per additional g/day, were associated with lower odds of being in a higher frailty category (q: 0.034, 0.034, and 0.038, respectively). No primary association in Model 2 remained significant after correction for multiple testing. Exploratory analyses identified quadratic relationships between the energy percentages from protein and polyunsaturated fatty acids and Montreal Cognitive Assessment (MoCA) scores (q = 0.0163 and 0.0111, respectively). **Conclusions**: Absolute intakes of certain fats were inversely associated with frailty, whereas the primary analyses of energy-adjusted dietary composition showed no significant associations after correction for multiple testing. These findings do not support recommending a specific macronutrient distribution. The exploratory cognitive findings require replication in longitudinal studies.

## 1. Introduction

Long COVID-19 is a heterogeneous clinical condition that can affect multiple dimensions of health. The World Health Organization (WHO) defines it as a condition occurring in individuals with a history of probable or confirmed severe acute respiratory syndrome coronavirus 2 (SARS-CoV-2) infection, usually three months after infection onset, with symptoms lasting at least two months that cannot be explained by an alternative diagnosis. Fatigue, dyspnea, and cognitive difficulties are among its characteristic manifestations [1]. Findings of muscular and metabolic abnormalities and changes in the gut microbiota support investigating nutritional factors associated with its clinical expression, although they do not demonstrate that inadequate intake causes these mechanisms [2,3].

The relationship between nutrition and the clinical manifestations of long COVID-19 may be bidirectional. Changes in taste and smell, loss of appetite, and gastrointestinal symptoms may impede eating, whereas insufficient intake could contribute to deterioration in nutritional and functional status. In a sample of 92 individuals receiving care after COVID-19, nutritional risk was accompanied by a higher frequency of certain symptoms and lower energy intake [4]. A subsequent clinical cohort also documented persistent nutritional problems and risk of sarcopenia during recovery [5]. These observations support dietary assessment but do not establish the direction of the associations. Evidence specifically addressing habitual macronutrient intake remains limited and heterogeneous. A study using seven-day food records in 46 patients with post-COVID-19 fatigue and 46 controls found no relevant differences in energy, protein, or carbohydrate intake that explained fatigue [6]. The CUME study, in turn, linked higher protein and lipid intakes to the subsequent presence of post-COVID-19 syndrome, a different question from their association with clinical severity among individuals already affected [7]. In 2026, a study of diet quality reported associations between poorer diet quality and greater symptom burden, particularly in women; however, its findings do not identify independent effects of specific macronutrients [8]. Nutritional trials likewise do not establish an optimal macronutrient composition for this condition. Favorable findings have been reported for L-arginine plus vitamin C and for creatine, whereas a small omega-3 trial detected no significant symptom improvements [9,10,11]. These interventions examine specific supplements and are not equivalent to modifying total protein intake or habitual fat distribution. The ReDIRECT trial found a modest improvement in participants’ selected primary symptom through a weight management program in individuals with overweight [12]. A 2026 feasibility trial evaluated nutritional counseling combined with physiotherapy without providing a conclusive estimate of the additional clinical benefit [13]. Taken together, these studies do not isolate the effect of individual macronutrients.

Previous analyses of the BioICOPER study have reported associations between macronutrient intake and selected vascular health parameters [14]. These findings do not establish their relationship with multidimensional symptom burden. Addressing this question requires distinguishing absolute intake from dietary composition: higher intake of a nutrient may simply reflect greater total energy intake [15]. Moreover, documented sex differences in the risk of long COVID-19 support considering sex and exploring stratified patterns without presupposing differences in nutritional associations [16].

Accordingly, this study aimed to examine associations between intakes of energy, protein, carbohydrates, fiber, total fat, and saturated fatty acids (SFA), monounsaturated fatty acids (MUFA), and polyunsaturated fatty acids (PUFA) and twelve clinical outcomes in adults with long COVID-19 from the BioICOPER study. The outcomes included global symptom burden, sleep quality, fatigue, memory failures, physical and mental quality of life, anxiety, depression, pain, frailty, dyspnea, and cognitive function. Both absolute intake and energy-adjusted dietary composition were evaluated, and sex-stratified correlations were explored. This approach aims to characterize nutritional correlates of clinical status and generate hypotheses for subsequent longitudinal studies and interventions.

## 2. Materials and Methods

### 2.1. Study Design and Participants

This observational study was a cross-sectional analysis of baseline data from adults with long COVID-19 enrolled in the BioICOPER cohort at the Primary Care Research Unit of Salamanca (APISAL). Each participant underwent a single baseline assessment, with all data collected within a two-week period; no longitudinal changes were examined in the present analysis. Although the protocol initially planned to include 300 participants [17], the cohort ultimately comprised 304 adults with long COVID-19. Patients were enrolled between 9 March 2023 and 10 September 2024. For the present study, 14 participants were excluded because they lacked a complete dietary record, leaving an analytical sample of 290 individuals: 89 men and 201 women. This sample was used to describe energy and macronutrient intake and examine its relationship with clinical manifestations. Association analyses used available cases for each outcome, without imputation of missing values. Recruitment began on 9 March 2023, after ethics committee approval had been obtained, to allow participant inclusion to progress within the project’s one-year funding period, which started on 1 January 2023. The study was subsequently registered at ClinicalTrials.gov on 18 April 2023 (NCT05819840), after recruitment had commenced. The study protocol has been published previously [17].

Participants were recruited consecutively from primary care and hospital settings. Adults who met the World Health Organization definition of long COVID-19 were eligible [1]. All participants provided written informed consent. Exclusion criteria were terminal illness, inability to travel to the health center or complete the assessment, a history of cardiovascular disease—including ischemic heart disease or cerebrovascular disease—and an estimated glomerular filtration rate below 30 mL/min/1.73 m^2^.

**Figure 1:**
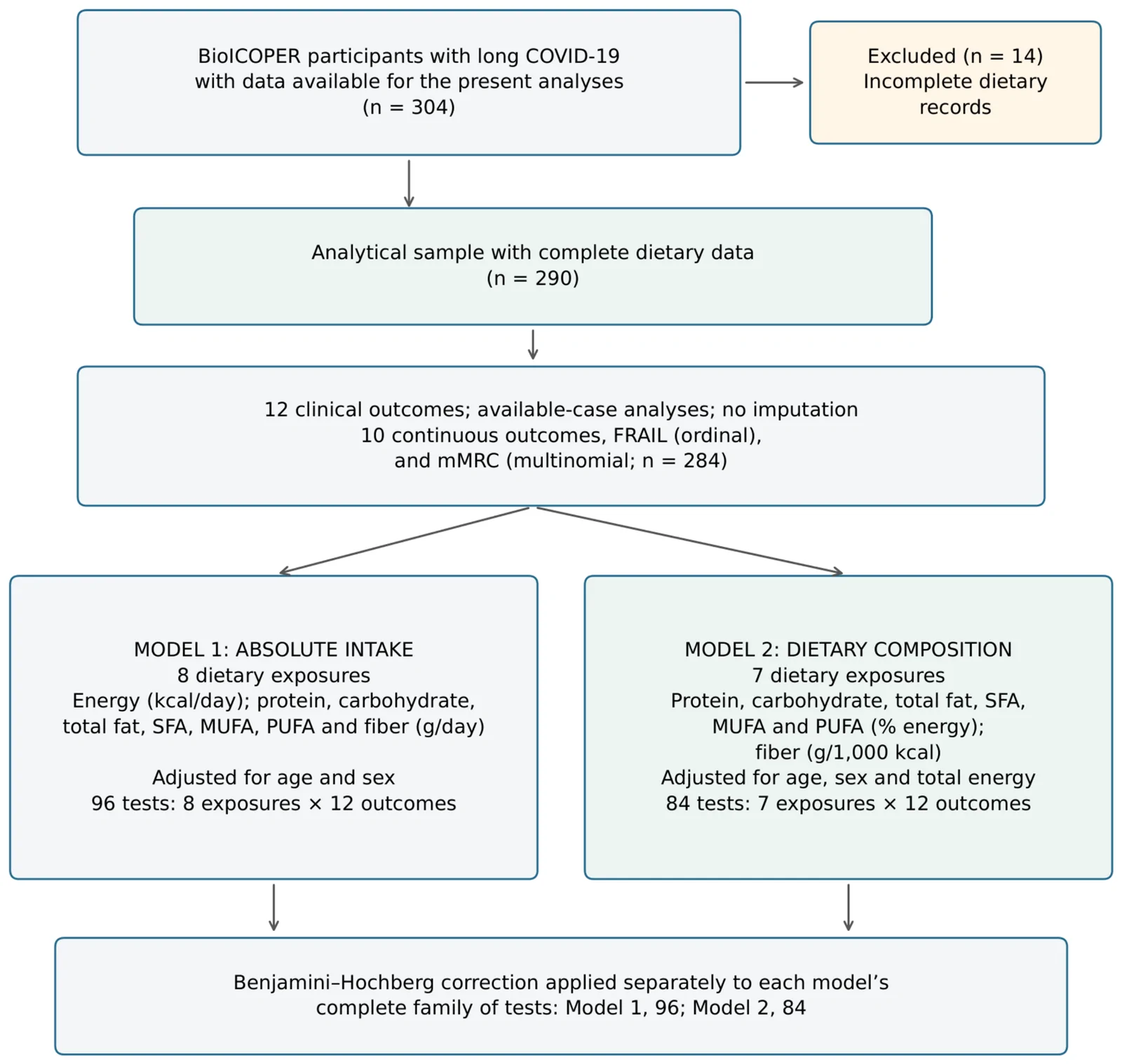
Of 304 participants with long COVID-19, 14 were excluded because of incomplete dietary records, leaving 290 participants in the analytical sample. Clinical outcomes were analyzed using available cases, without imputation. SFA, saturated fatty acids; MUFA, monounsaturated fatty acids; PUFA, polyunsaturated fatty acids; FRAIL, fatigue, resistance, ambulation, illnesses, and loss of weight; mMRC, modified Medical Research Council dyspnea scale.

Participant selection, exclusions due to incomplete dietary information, and the composition of the analytical sample are presented in Figure 1. The study was reported in accordance with the Strengthening the Reporting of Observational Studies in Epidemiology (STROBE) recommendations for observational studies [18].

### 2.2. Dietary Intake Assessment

Dietary intake was assessed using a food and beverage record completed over seven consecutive days with the EVIDENT mobile application [19]. Record completeness was checked by verifying that each participant had food and beverage entries for all seven consecutive assessment days. This tool was developed and validated by the Primary Care Research Group of Castilla y León, a member of the Research Network on Preventive Activities and Health Promotion (REDIAPP), and is registered under intellectual property number 00/2014/2207. Participants recorded the foods and beverages consumed and their portion sizes daily, classifying them into the application’s predefined groups. Energy intake and intakes of protein, carbohydrates, fiber, total fat, and saturated, monounsaturated, and polyunsaturated fatty acids were estimated using the Spanish food composition tables incorporated into EVIDENT [19].

All data collection was completed within a two-week period for each participant. Dietary intake was recorded prospectively during the seven consecutive days immediately following study inclusion. Dietary and clinical assessments were therefore conducted in close temporal proximity, although some clinical questionnaires referred to symptoms experienced during the preceding weeks.

Mean daily intakes were calculated by dividing the cumulative amounts recorded over the assessment period by seven. Energy was expressed in kilocalories per day and nutrients in grams per day. To characterize dietary composition, energy contributions from protein and carbohydrates were calculated using a conversion factor of 4 kcal/g, and those from total fat and the different fatty acid classes using a factor of 9 kcal/g. The percentage of energy supplied by each nutrient was obtained by dividing its energy contribution by total recorded energy intake and multiplying by 100. Fiber density, expressed in grams per 1000 kcal, was calculated by dividing daily fiber intake by total daily energy intake and multiplying by 1000.

Attainment of five population-level dietary targets was assessed in the 290 participants with complete dietary records: protein, 10–15% of total energy, in accordance with the distribution target proposed by WHO and the Food and Agriculture Organization of the United Nations (FAO) [20]; carbohydrates, 45–60% [21]; total fat, 20–35% [22]; saturated fatty acids, below 10% [23]; and dietary fiber, at least 25 g/day [21]. Both bounds were included in the intervals for protein, carbohydrates, and total fat; the criterion < 10% was applied strictly for saturated fatty acids. The criteria and their sources are detailed in Table S1. Each target was evaluated independently, without constructing a composite diet quality score. Attainment was described using absolute frequencies and percentages, with 95% confidence intervals (CIs) calculated using the Wilson method, both overall and by sex. Proportions in men and women were compared using two-sided Fisher’s exact tests, and the Benjamini–Hochberg (BH) procedure was applied to these five comparisons.

These targets were used solely for descriptive classification of intake and are not diagnostic criteria for individual nutritional deficiency or excess. In particular, obtaining more than 15% of energy from protein does not imply excessive or harmful intake. Individual protein adequacy in grams per kilogram of body weight per day was not assessed.

### 2.3. Clinical Variables and Multidimensional Symptom Assessment

Clinical information was collected through medical record review, a structured interview, and validated questionnaires, in accordance with the BioICOPER protocol [17]. Information was collected on the history of SARS-CoV-2 infection, the number and dates of episodes, the diagnostic procedure, hospitalization during the acute phase, and the time between infection and assessment.

Persistent manifestations were organized into six clinical domains: systemic—fatigue, lack of energy, and fever; neurocognitive—memory loss, concentration difficulties, and brain fog or confusion; respiratory or cardiovascular—dyspnea, cough, sore throat, and weight loss; musculoskeletal—muscle pain, joint pain, and limited mobility; neurological or neuromuscular—headache, changes in taste or smell, and reduced reflexes; and psychosocial or psychiatric—depressive symptoms, anxiety, and sleep disorders [17]. Global symptom burden was calculated as the number of domains with at least one persistent manifestation, yielding a score from 0 to 6. Thus, this indicator reflects the extent of involvement across domains rather than the total number or intensity of symptoms.

The impact of fatigue over the preceding four weeks was assessed using the Modified Fatigue Impact Scale (MFIS) [24]. Its 21 items are scored from 0 to 4 and cover physical, cognitive, and psychosocial dimensions. The total score, ranging from 0 to 84, was used in this study; higher values indicate a greater impact of fatigue.

Sleep quality over the preceding month was assessed using the Spanish version of the Pittsburgh Sleep Quality Index (PSQI) [25,26]. This instrument comprises seven components: subjective sleep quality, sleep latency, duration, habitual sleep efficiency, sleep disturbances, use of sleep medication, and daytime dysfunction. The global score ranges from 0 to 21, with higher scores indicating poorer sleep quality.

Perceived memory failures in daily life were assessed using the 30-item Memory Failures of Everyday questionnaire (MFE-30), validated in the Spanish population [27]. Responses indicate the frequency of these difficulties across five categories, from ‘never or almost never’ to ‘always or almost always’. The total score was used, with higher values indicating more frequent subjective memory failures. Functional limitation attributable to dyspnea was assessed using the modified Medical Research Council (mMRC) scale [28]. Its five grades, from 0 to 4, range from breathlessness only during strenuous exercise to breathlessness that prevents leaving the home or occurs during basic activities such as dressing. Higher scores indicate greater limitation.

Pain was assessed using the Spanish version of the Graded Chronic Pain Scale, also known as the Chronic Pain Grade Questionnaire [29]. The questionnaire comprises eight items that include the persistence of pain, its current, maximum and average intensity, and the associated limitation and interference in daily, work, social and leisure activities. For this analysis, the total score was used, calculated by adding items 2 to 8, scored from 0 to 10, with a theoretical range of 0 to 70 points. The item referring to days of activity limitation during the preceding three months was coded into eleven categories, from 0 to 10. The first item, which records the number of days with pain during the preceding six months, was not included in the total. The score was analyzed as a continuous variable, with higher values indicating greater pain-related impairment.

Health-related quality of life was measured using the Spanish version of the Short Form-36 Health Survey (SF-36) [30]. Its 36 items cover eight dimensions: physical functioning, role limitations due to physical health, bodily pain, general health, vitality, social functioning, role limitations due to emotional problems, and mental health. The Physical Component Summary (PCS) and Mental Component Summary (MCS) were analyzed in this study. For both, higher scores indicate better health status.

Frailty was assessed using the FRAIL (fatigue, resistance, ambulation, illnesses, and loss of weight) scale [31], which comprises five dichotomous items addressing fatigue, resistance, ambulation, illnesses, and weight loss. Its total score ranges from 0 to 5 and distinguishes robust (0 points), prefrail (1–2 points), and frail (3–5 points) individuals. The original score was retained for descriptive analyses; categories of 0, 1, 2, and ≥3 points were used in the regression models.

Anxiety and depressive symptoms were assessed using the Spanish version of the Goldberg Anxiety and Depression Scale (GADS) [32,33]. Each of its two subscales contains nine dichotomous items, with one point assigned to each affirmative response. The original anxiety and depression scores were used in the analyses, without applying cutoffs or dichotomizing outcomes. Higher scores indicate more symptoms in the corresponding domain.

Cognitive function was examined using the validated Spanish version of the Montreal Cognitive Assessment (MoCA) [34,35]. This test assesses attention and concentration, executive function, memory, language, visuoconstructional skills, conceptual reasoning, calculation, and orientation. The total score was used as a continuous variable, with a maximum of 30 points; higher scores indicate better cognitive performance. The MoCA scores used in all analyses incorporated the education-related correction.

Twelve clinical outcomes were analyzed: global symptom burden, PSQI, MFIS, MFE-30, PCS, MCS, GADS anxiety and depression, pain, FRAIL, mMRC, and MoCA. Ordinal variables and continuous variables with non-normal distributions were described using medians and interquartile ranges. For regression analyses, mMRC scores were grouped into categories of 0, 1, 2, and ≥3; model specifications are detailed in Section 2.5. Higher scores for global symptom burden, PSQI, MFIS, MFE-30, FRAIL, GADS, pain, and mMRC indicate poorer clinical status, whereas higher PCS, MCS, and MoCA scores indicate better outcomes.

### 2.4. Covariates and Other Study Variables

Age, sex, body mass index (BMI), alcohol consumption, hospitalization during the acute phase of COVID-19, and time since the infection associated with long COVID-19 onset were recorded using the standardized procedures of the BioICOPER protocol [17]. These variables were used to describe sample characteristics.

Age and sex were included as adjustment covariates in the regression models because of their clinical relevance and potential relationships with both dietary intake and the outcomes assessed. Model 1 was adjusted for both variables, whereas Model 2 additionally included total energy intake.

### 2.5. Statistical Analysis

Analyses included the 290 participants with long COVID-19 who had a complete seven-day dietary record. Each analysis used participants with available information for the variables involved, without imputation of missing data; therefore, sample size varied by clinical outcome. All tests were two-sided. Analyses were performed using IBM SPSS Statistics, version 32 (IBM Corp., Armonk, NY, USA). Continuous variable distributions were examined using the Shapiro–Wilk test together with inspection of histograms, normal quantile plots, and box plots. Approximately normally distributed variables were described using means and standard deviations (SDs), whereas ordinal or non-normally distributed variables were summarized using medians and interquartile ranges. Categorical variables were presented as absolute frequencies and percentages. The marginal distributions of the outcomes were not used as the sole criterion for assessing the suitability of regression models. Sample characteristics and dietary intake were described overall and by sex. Comparisons between men and women used the Mann–Whitney U test for continuous or ordinal variables and Pearson’s chi-square test or two-sided Fisher’s exact test for categorical variables, as appropriate. Characteristics of participants with complete dietary records were also compared with those of participants excluded because of incomplete dietary information. These descriptive comparisons were reported with nominal *p* values, without correction for multiple testing. The five sex comparisons of dietary target attainment used two-sided Fisher’s exact tests and Benjamini–Hochberg correction, as described in the dietary assessment section.

Unadjusted associations between the eight dietary exposures expressed in absolute units and the twelve clinical outcomes were explored using Spearman’s correlation coefficient (ρ). In the overall sample, the Benjamini–Hochberg procedure was applied to the family of 96 correlations—eight exposures by twelve outcomes. Sex-stratified correlations were also calculated; these were considered exploratory and displayed with nominal *p* values. These analyses were not interpreted as formal tests of differences between men and women. For adjusted associations, a separate model was fitted for each combination of dietary exposure and clinical outcome. Two specifications were used. Model 1 included eight exposures: total energy, expressed in kcal/day, and protein, carbohydrates, fiber, total fat, and saturated, monounsaturated, and polyunsaturated fatty acids, expressed in g/day. Each model was adjusted for age and sex, yielding 96 association tests. Model 2 examined seven exposures: protein, carbohydrates, total fat, and saturated, monounsaturated, and polyunsaturated fatty acids, expressed as percentages of total energy, and fiber, expressed in g/1000 kcal. Each exposure was analyzed separately, adjusting for age, sex, and total energy intake, yielding 84 tests. These specifications examined associations with absolute intake and dietary composition, respectively. The models were not interpreted as estimates of substitution between specific nutrients. Ten outcomes—global symptom burden, PSQI, MFIS, MFE-30, the physical and mental components of the SF-36, GADS anxiety and depression, pain, and MoCA—were treated as continuous variables and analyzed using ordinary least squares linear regression with heteroscedasticity-consistent standard errors, type 3 (HC3). Estimates were reported as unstandardized regression coefficients (B) and 95% confidence intervals. For exposures expressed as energy percentages, coefficients correspond to a one-percentage-point increase; for fiber in Model 2, they correspond to a 1-g/1000-kcal increase. When figures used standardized estimates or one-standard-deviation increments in exposure, the corresponding scale was specified in the figure legend. FRAIL scores were grouped into four categories—0, 1, 2, and ≥3 points—and analyzed using proportional-odds ordinal logistic regression. Odds ratios (ORs) and 95% confidence intervals were estimated; an OR below 1 indicates lower odds of being in a higher frailty category. The proportional-odds assumption was examined using the test of parallel lines. mMRC scores were likewise grouped into categories of 0, 1, 2, and ≥3 and analyzed using multinomial logistic regression, with category 0 as the reference. For each exposure, a global likelihood-ratio test with three degrees of freedom compared the model containing the exposure and covariates with the model containing covariates alone. This global test, rather than individual between-category contrasts, was used in the primary correction for multiple testing. Nominal *p* values and Benjamini–Hochberg-adjusted q values were reported. Correction was applied separately to the 96 tests in Model 1 and the 84 tests in Model 2, with one test for each exposure–outcome combination. Regression test families were kept separate from those for correlations and dietary target attainment. Within corrected families, q < 0.05 was considered statistical evidence; associations with *p* < 0.05 and q ≥ 0.05 were identified as nominal and exploratory. The functional specification of the 70 linear regression models in Model 2 was examined using the classical Ramsey regression equation specification error test (RESET). The squared and cubed standardized fitted values were added jointly, and their contribution was assessed using a conventional F test. Diagnostic *p* values were interpreted exploratorily, without correction for multiple testing. As an additional exploratory analysis, potential quadratic relationships between MoCA scores and the seven Model 2 dietary exposures were evaluated, retaining adjustment for age, sex, and total energy intake. In the quadratic models, confidence intervals and two-sided *p* values were calculated using HC3 robust standard errors and a t distribution with 280 residual degrees of freedom. Benjamini–Hochberg correction was applied separately to the seven tests of quadratic terms, using unrounded *p* values. These tests formed an independent exploratory family and were not included in the 84 primary model tests. Quadratic relationships were displayed using adjusted predictions and pointwise 95% confidence bands based on the HC3 robust estimator, over the range between the 5th and 95th percentiles of each exposure. *p* and q values were generally reported to three decimal places and as <0.001 when below that threshold. Additional decimal places were retained when necessary to interpret diagnostics or the correction for multiple testing.

As a sensitivity analysis, associations of total fat, SFA, MUFA, and PUFA intake with FRAIL categories (0, 1, 2, and ≥3) were evaluated using separate ordinal logistic regression models, retaining each exposure in g/day and adjusting for age, sex, and total energy intake. The proportional odds assumption was assessed using the test of parallel lines. These analyses were considered exploratory, and *p*-values were reported without additional correction for multiple testing.

### 2.6. Ethical Considerations

The project was approved by the Research Ethics Committee for Medicines of the Salamanca Health Area on 27 June 2022 (CEIm; reference PI 2022 06 1048). The study was conducted in accordance with the ethical principles of the Declaration of Helsinki. Confidentiality and personal data processing complied with Spanish Organic Law 3/2018 and Regulation (EU) 2016/679. Before enrollment, all participants received detailed information on the study objectives and procedures and provided written informed consent.

### 2.7. Use of Artificial Intelligence Tools

During manuscript preparation, ChatGPT (GPT-6, OpenAI, https://chatgpt.com) was used to support language editing, text organization and coherence, code development, and the preparation and adaptation of figures based on the study results. Artificial intelligence was not used to generate participant data or fabricate results. The authors verified the content and graphical representations against the original data and statistical outputs, reviewed and approved the final version, and take full responsibility for the manuscript’s content.

## 3. Results

### 3.1. General Characteristics

Among the variables assessed in Table S2, no statistically significant departure from normality was detected for age or the energy percentages from total fat, saturated fatty acids, and monounsaturated fatty acids. The remaining quantitative variables included in that table showed departures from normality. Sex and hospitalization were treated as categorical variables and were not subjected to normality tests.

Participants without complete macronutrient records were younger than those with complete records (median age: 44.5 versus 53.0 years; *p* = 0.003); no other significant differences were observed in the sociodemographic and clinical variables analyzed in this study (Table S3).

Table 1 presents the sociodemographic and clinical characteristics of the 290 participants included in the analysis, overall and by sex. Men were older and had higher BMI, alcohol consumption, and frequency of hospitalization during acute COVID-19. Women had higher global symptom, sleep, fatigue, anxiety, depression, pain, and frailty scores; men had higher SF-36 physical and mental component scores. A sex difference was also observed in the distribution of mMRC scores. FRAIL categories 0, 1, 2, and ≥3 points included 105 (36.2%), 109 (37.6%), 56 (19.3%), and 20 participants (6.9%), respectively. Among the 284 participants with available dyspnea information, mMRC categories 0, 1, 2, and ≥3 included 69 (24.3%), 151 (53.2%), 45 (15.8%), and 19 participants (6.7%), respectively.

Table 2 presents daily energy and nutrient intakes, macronutrient energy percentages, and fiber density, overall and by sex. In nominal comparisons, men had higher absolute saturated fatty acid intake (*p* = 0.035), whereas women had a higher energy percentage from polyunsaturated fatty acids (*p* < 0.001). These descriptive comparisons were not corrected for multiple testing.

**Figure 2:**
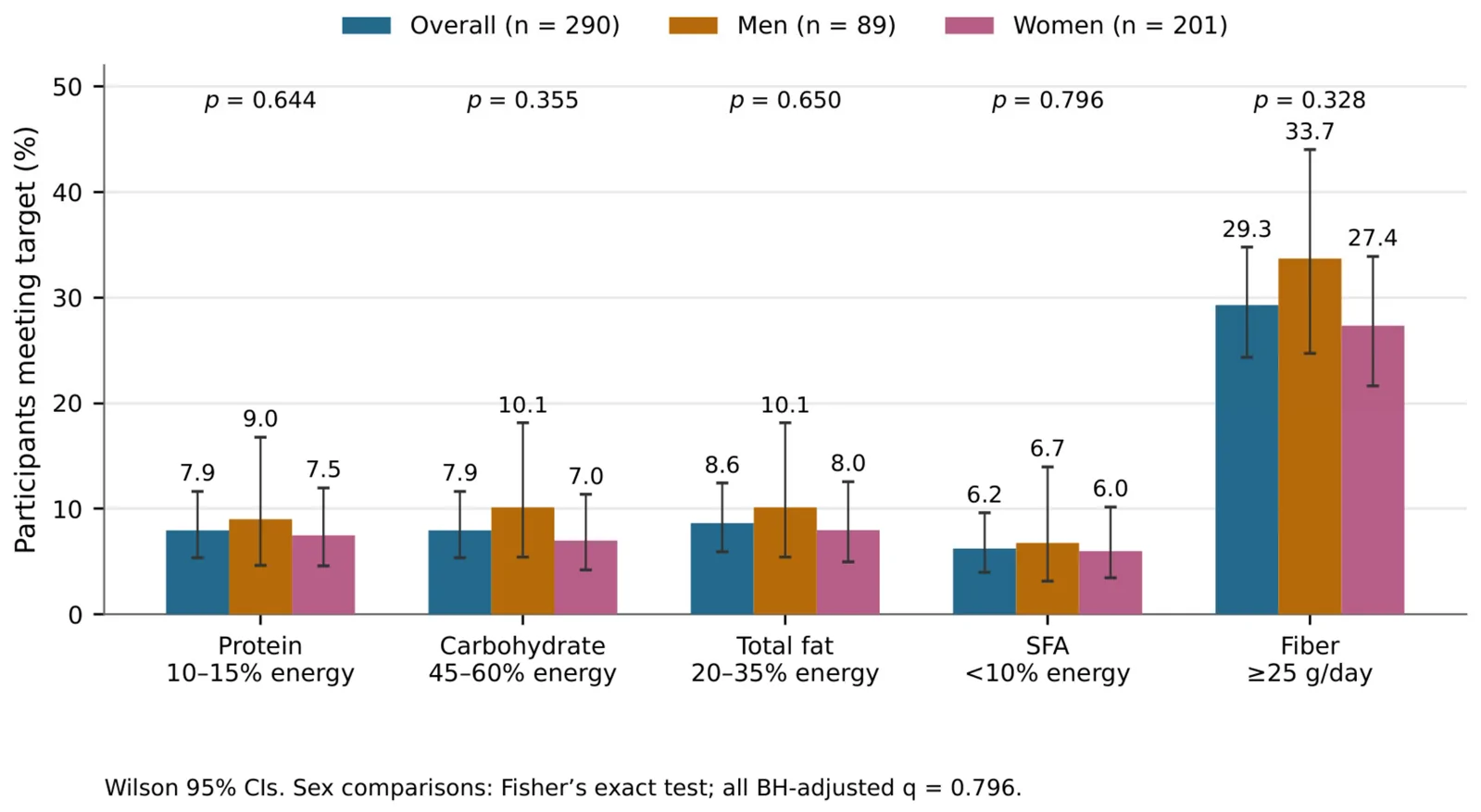
Attainment of selected dietary targets overall and by sex. Bars show the percentage of participants meeting each target, with Wilson 95% confidence intervals. Blue, overall sample (N = 290); amber, men (n = 89); pink, women (n = 201). *p* values above each group of bars are from two-sided Fisher exact tests comparing men with women. Targets: protein 10–15 E%, carbohydrate 45–60 E%, total fat 20–35 E%, saturated fatty acids < 10 E%, and dietary fiber ≥ 25 g/day. All five sex comparisons had q = 0.796 after Benjamini–Hochberg correction. SFA, saturated fatty acids; E%, percentage of total reported daily energy intake; CI, confidence interval. These operational targets do not constitute a validated composite index of diet quality.

Among the 290 participants, 23 (7.9%) met the selected protein distribution target, 23 (7.9%) the carbohydrate target, 25 (8.6%) the total fat target, 18 (6.2%) the saturated fatty acid target, and 85 (29.3%) the dietary fiber target. The proportion meeting each target did not differ significantly between men and women (two-sided Fisher’s exact tests: *p* = 0.328–0.796; all Benjamini–Hochberg q values = 0.796). Detailed counts and confidence intervals are presented in Table S4 and Figure 2. The low proportion within the selected protein interval indicates non-attainment of that distribution target and does not establish protein deficiency.

### 3.2. Association Analysis

**Figure 3:**
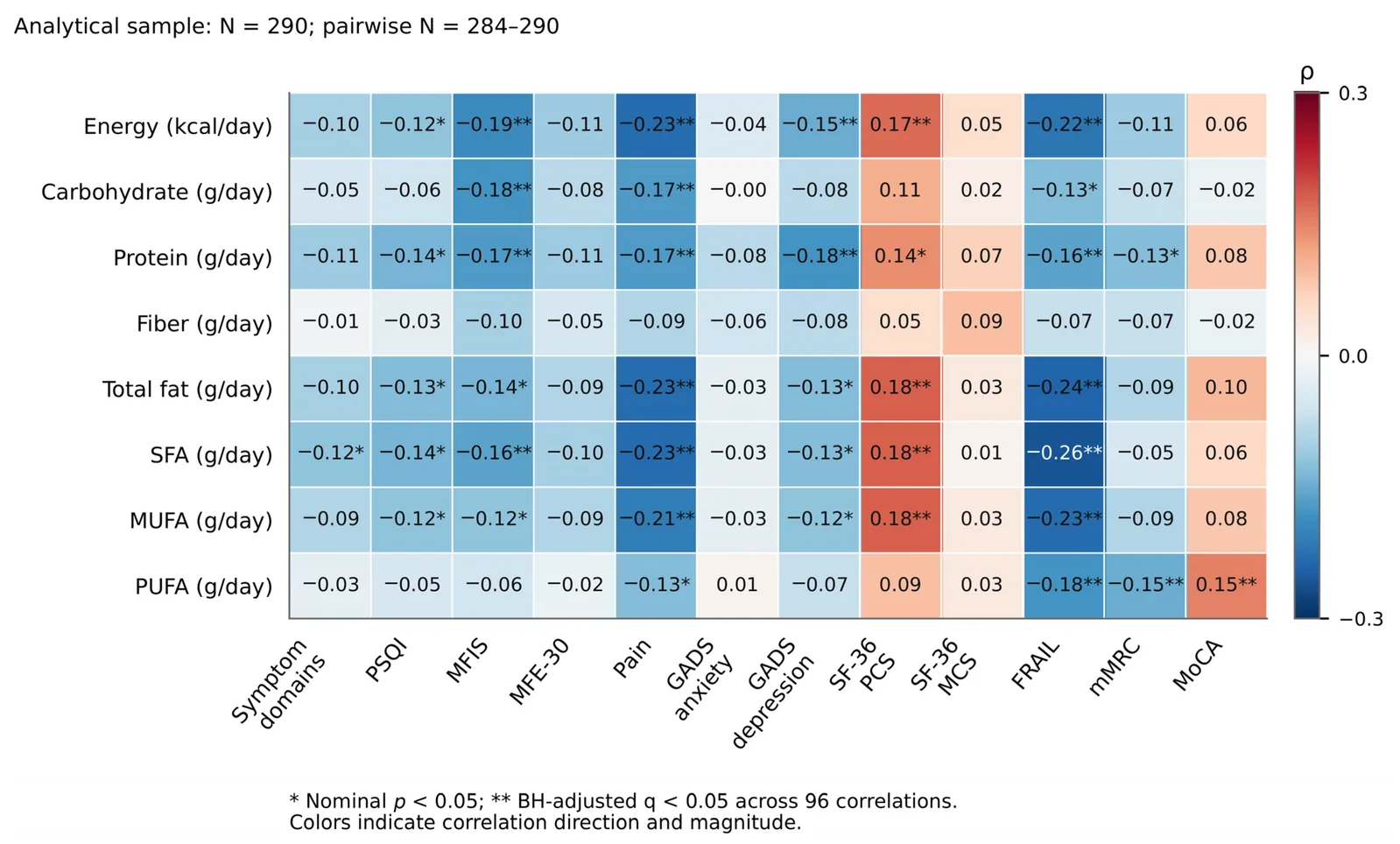
Spearman correlations between daily dietary intake and clinical characteristics in the overall analytical sample (N = 290). Cell values represent Spearman’s ρ. * *p* < 0.05, nominal; ** q < 0.05 after Benjamini–Hochberg correction for multiple testing, across 96 correlations. Blue indicates negative correlations, red indicates positive correlations, and white indicates zero. SFA, saturated fatty acids; MUFA, monounsaturated fatty acids; PUFA, polyunsaturated fatty acids; PSQI, Pittsburgh Sleep Quality Index; MFIS, Modified Fatigue Impact Scale; MFE-30, 30-item Memory Failures of Everyday questionnaire; GADS, Goldberg Anxiety and Depression Scale; SF-36, 36-Item Short Form Health Survey; PCS, physical component summary; MCS, mental component summary; FRAIL, frailty scale; mMRC, modified Medical Research Council dyspnea scale; MoCA, Montreal Cognitive Assessment.

Figure 3 shows correlations between daily intake and clinical characteristics in the overall analytical sample (N = 290; pairwise N = 284–290; Spearman’s ρ range: −0.26 to 0.18). After Benjamini–Hochberg correction across the 96 correlations (q < 0.05), energy intake was negatively correlated with fatigue (MFIS; ρ = −0.19), pain (ρ = −0.23), frailty (ρ = −0.22), and depressive symptoms (GADS; ρ = −0.15), and positively correlated with the SF-36 Physical Component Summary (PCS; ρ = 0.17). Carbohydrate intake was negatively correlated with fatigue (ρ = −0.18) and pain (ρ = −0.17). Protein intake was negatively correlated with fatigue (ρ = −0.17), pain (ρ = −0.17), depressive symptoms (GADS depression subscale; ρ = −0.18), and frailty (ρ = −0.16). Total fat, SFA, and MUFA were negatively correlated with pain (ρ = −0.23, −0.23, and −0.21, respectively) and frailty (ρ = −0.24, −0.26, and −0.23, respectively), and positively correlated with SF-36 PCS (ρ = 0.18, 0.18, and 0.18, respectively). SFA were also negatively correlated with fatigue (ρ = −0.16). PUFA were negatively correlated with frailty (ρ = −0.18) and dyspnea (mMRC; ρ = −0.15), and positively correlated with MoCA scores (ρ = 0.15). No associations remained significant after BH correction for fiber intake, global symptom burden, sleep quality (PSQI), anxiety symptoms, MFE-30 scores, or the SF-36 Mental Component Summary. Other nominal associations shown in the figure did not remain significant after correction.

Supplementary Figure S1 shows sex-stratified Spearman correlations. In men (n = 89), nominal negative correlations were observed between intakes of energy, protein, total fat, SFA, MUFA, and PUFA and frailty. Intakes of energy, protein, total fat, MUFA, and PUFA also showed nominal negative correlations with mMRC dyspnea scores. PUFA intake was positively correlated with MoCA scores. In women (n = 201), energy and carbohydrate intakes were inversely correlated with MFIS and pain; total fat, SFA, and MUFA were also inversely correlated with pain. Fiber was inversely correlated with MFE-30. Energy, total fat, and MUFA were positively correlated with the SF-36 physical component, whereas energy, total fat, SFA, MUFA, and PUFA were inversely correlated with frailty. These associations are presented with nominal significance and without correction for multiple comparisons, consistent with the exploratory nature of Figure S1. The figure alone does not establish that correlations differ by sex; this would require a formal interaction test.

**Figure 4:**
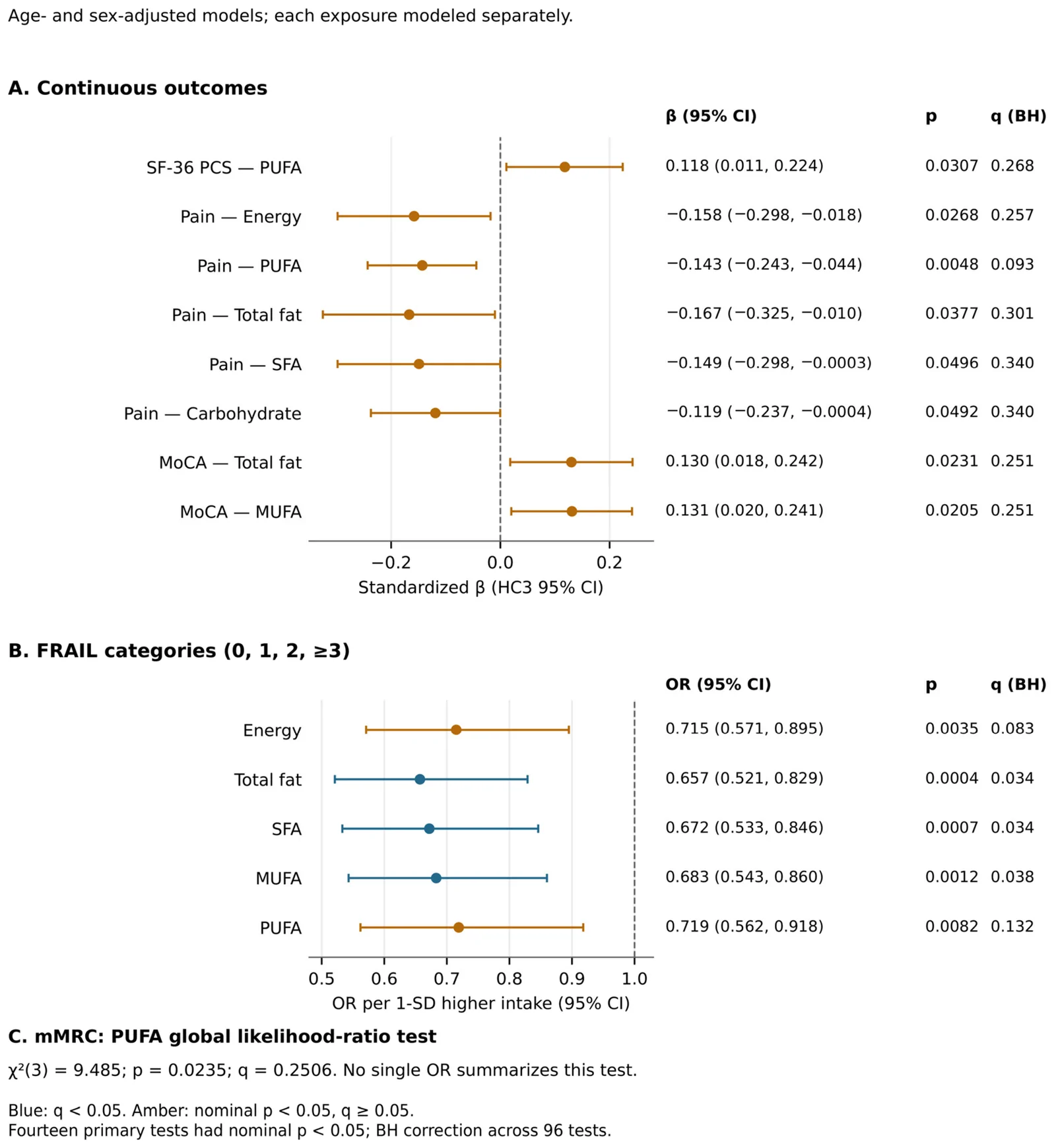
Nominal associations between absolute dietary intake and clinical outcomes in Model 1. Only associations with nominal *p* < 0.05 are shown. Continuous outcomes are presented as standardized β coefficients (B × SD of exposure/SD of outcome) and HC3 robust 95% confidence intervals (CIs) from separate linear regression models adjusted for age and sex. FRAIL associations are presented as odds ratios (ORs) and 95% CIs per 1-SD higher nutrient intake from separate proportional-odds ordinal logistic regression models. For mMRC, the PUFA association is presented as a global likelihood-ratio test (χ^2^(3) = 9.485; *p* = 0.0235; q = 0.2506), as the multinomial model does not provide a single overall OR. Orange indicates nominal significance without significance after Benjamini–Hochberg (BH) correction; blue indicates q < 0.05 after correction across 96 primary tests. Sample sizes varied by outcome (N = 284–290). ORs below 1 indicate lower odds of being in a higher FRAIL category. PUFA, polyunsaturated fatty acids; SFA, saturated fatty acids; MUFA, monounsaturated fatty acids. HC3, heteroscedasticity-consistent covariance estimator, type 3; SD, standard deviation; PCS, physical component summary; SF-36, 36-Item Short Form Health Survey; MoCA, Montreal Cognitive Assessment; FRAIL, fatigue, resistance, ambulation, illnesses, and loss of weight; mMRC, modified Medical Research Council dyspnea scale.

**Figure 5:**
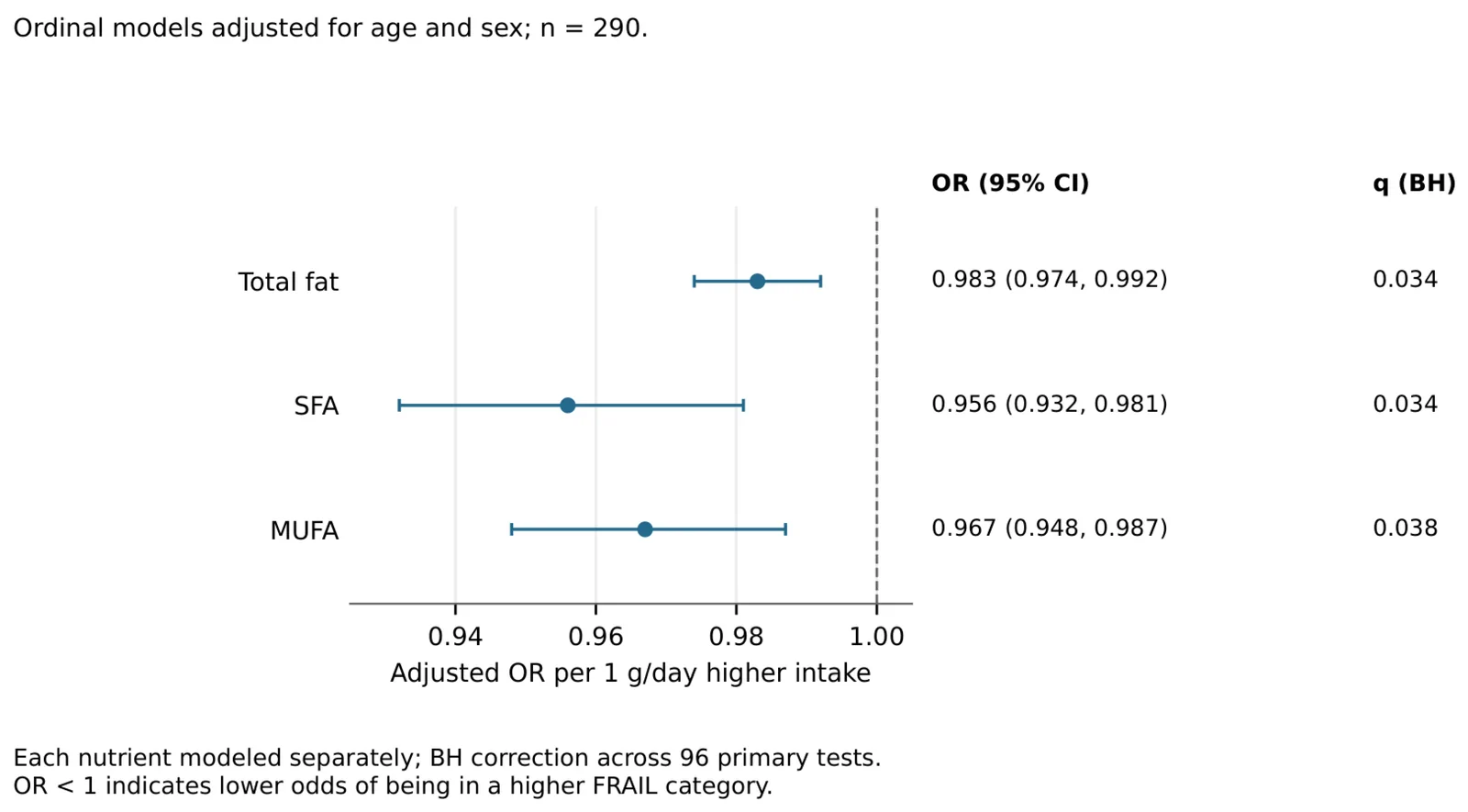
Adjusted odds ratios (ORs) and 95% confidence intervals (CIs) for the association between daily fat intake and FRAIL category. Each nutrient was evaluated in a separate proportional-odds ordinal logistic regression model adjusted for age and sex (n = 290); estimates are per 1-g/day increase. The dashed vertical line indicates the null value (OR = 1). q values were adjusted using the Benjamini–Hochberg procedure across the 96 primary tests. ORs below 1 indicate lower odds of being in a higher FRAIL category. SFA, saturated fatty acids; MUFA, monounsaturated fatty acids. FRAIL, fatigue, resistance, ambulation, illnesses, and loss of weight.

Table S5 presents the 96 associations between the eight dietary exposures and the 12 clinical outcomes in Model 1, with nominal *p* values and Benjamini–Hochberg correction. Fourteen associations had nominal *p* values < 0.05 (Figure 4). Only three remained significant after Benjamini–Hochberg correction (Figure 5). All three involved FRAIL scores grouped into four categories. In age- and sex-adjusted ordinal models, higher daily intakes of total fat (OR = 0.983; 95% CI: 0.974–0.992; *p* = 0.0004; q = 0.034), saturated fatty acids (OR = 0.956; 95% CI: 0.932–0.981; *p* = 0.0007; q = 0.034), and monounsaturated fatty acids (OR = 0.967; 95% CI: 0.948–0.987; *p* = 0.0012; q = 0.038) were associated with lower odds of being in a higher FRAIL category per additional g/day of intake. These associations were estimated in separate models and should not be interpreted as causal effects or as effects independent of other macronutrients (Figure 5). No association with a continuous outcome remained significant after correction. No evidence of violation of the proportional-odds assumption was detected in the three age- and sex-adjusted frailty models for total fat (χ^2^ = 2.440; 6 df; *p* = 0.875), saturated fatty acids (χ^2^ = 2.514; 6 df; *p* = 0.867), and monounsaturated fatty acids (χ^2^ = 2.016; 6 df; *p* = 0.918). For mMRC, the global association with PUFA was nominal (χ^2^(3) = 9.485; *p* = 0.023; q = 0.251) and did not survive correction. The mMRC category 2 versus 0 contrast for PUFA was also nominal (OR = 0.867 per additional g/day; 95% CI: 0.775–0.969; *p* = 0.012) and should therefore be considered exploratory.

In sensitivity analyses additionally adjusted for total energy intake (n = 290), total fat intake retained a borderline nominal inverse association with higher FRAIL categories (OR per 1 g/day: 0.9787; 95% CI: 0.9580–0.9998; *p* = 0.048). Associations with SFA, MUFA, and PUFA did not reach nominal statistical significance (*p* = 0.091, 0.177, and 0.321, respectively). Tests of parallel lines showed no evidence of violation of the proportional odds assumption (all *p* ≥ 0.849; Table S8).

Table S6 presents the 84 adjusted models in Model 2, examining associations between seven intake variables and 12 clinical outcomes, and Figure S2 shows the two nominal associations. After adjustment for age, sex, and total energy intake, two nominal associations with MoCA scores were observed: an inverse association with the energy percentage from carbohydrates (B = −0.082; 95% CI: −0.148 to −0.016; *p* = 0.015) and a positive association with the energy percentage from total fat (B = 0.079; 95% CI: 0.002 to 0.155; *p* = 0.043) (Figure S2). Neither association remained significant after Benjamini–Hochberg correction (both q = 0.861). The classical Ramsey RESET detected no evidence of functional misspecification in the 70 linear models in Model 2 (*p* = 0.054–0.998). In the seven MoCA models, *p* values ranged from 0.054 to 0.352. These diagnostics were interpreted exploratorily.

In exploratory analyses, the quadratic terms for the energy percentages from protein (B = −0.0563; HC3 robust 95% CI: −0.0952 to −0.0175; *p* = 0.0046; q = 0.0163) and polyunsaturated fatty acids (B = −0.1214; 95% CI: −0.1964 to −0.0465; *p* = 0.0016; q = 0.0111) were associated with MoCA scores. The protein curve was concave, with higher predicted MoCA scores at intermediate intake levels; for PUFA, predicted scores increased and tended to flatten at higher levels. Both models were adjusted for age, sex, and total energy intake and included 286 participants. None of the other five quadratic terms was significant after Benjamini–Hochberg correction (Figure S3 and Table S7).

To illustrate the magnitude of these associations, holding age, sex, and total energy intake constant, the protein model predicted a MoCA score approximately 0.83 points higher at 19% than at 15% of total energy, and 0.56 points lower at 22% than at 19%. For PUFA, the predicted score was approximately 1.72 points higher at 7.5% than at 4.5% of total energy. These illustrative comparisons were selected within the exposure ranges displayed in Figure S3 and were calculated from the coefficients reported in Table S7.

## 4. Discussion

### 4.1. Main Study Findings

In this cross-sectional analysis of 290 adults with long COVID-19 from the BioICOPER cohort, associations between macronutrient intake and clinical status were limited and depended on how dietary exposure was expressed. In age- and sex-adjusted models, higher absolute intakes of total fat and saturated and monounsaturated fatty acids were associated with lower odds of being in a higher frailty category. These three associations remained significant after Benjamini–Hochberg correction. However, none of the primary associations with dietary composition, expressed as energy percentages or fiber density and additionally adjusted for total energy intake, remained significant after correction for multiple comparisons. The findings therefore do not identify a macronutrient distribution consistently associated with a better overall clinical profile.

The distinction between the two specifications is relevant. Absolute intake of a nutrient may reflect not only its specific consumption but also the total amount of food and energy consumed [15]. One possible interpretation is that participants with less frailty retained a greater ability to eat, shop for food, or prepare meals. However, because exposure units and energy adjustment changed simultaneously, the findings cannot attribute the difference between models exclusively to energy or demonstrate reverse causation. Moreover, saturated and monounsaturated fatty acids are components of total fat; their associations arise from related exposures and do not constitute three independent confirmations of a beneficial effect.

Additional adjustment for total energy intake in models retaining fat intake in g/day reduced the statistical evidence for associations with FRAIL, although the estimates remained inverse. Only total fat retained a borderline nominal association, and the estimates were less precise, supporting a cautious interpretation (Table S8).

Sensitivity analyses suggested nonlinear relationships between MoCA scores and the energy percentages from protein and polyunsaturated fatty acids, with q values of 0.0163 and 0.0111, respectively, after correction across the seven quadratic terms assessed. These observations are exploratory and do not establish optimal intake thresholds. Likewise, the low attainment of selected dietary targets—6.2% had saturated fat intake below 10% of energy, and 29.3% reached 25 g of fiber daily—describes opportunities to improve diet without demonstrating that these characteristics explain symptom persistence or intensity.

Although the quadratic terms remained statistically significant after correction for multiple testing, the clinical relevance of the predicted MoCA score differences remains uncertain. These estimates describe cross-sectional differences in expected cognitive test performance and should not be interpreted as within-person cognitive improvement or deterioration. The findings therefore do not establish a clinically beneficial intake range or support recommending specific protein or PUFA proportions to improve cognition.

### 4.2. Comparison with Previous Studies

Evidence on diet and long COVID-19 is heterogeneous in terms of populations, exposures, and outcomes. Studies jointly evaluating multiple clinical dimensions and macronutrient intake remain limited, although several have examined specific aspects. Deer et al. observed nutritional risk in a high proportion of 92 individuals receiving care after COVID-19, accompanied by lower energy intake and a higher frequency of certain symptoms [4]. In a cohort of 222 individuals receiving care from primary care dietitians, Slotegraaf et al. documented nutritional problems and risk of sarcopenia, even in a population with a high prevalence of overweight and obesity [5]. These studies support nutritional assessment but do not establish that insufficient intake causes symptoms. Improvements during follow-up in the second cohort likewise cannot be attributed solely to dietary treatment, given the absence of a control group. In contrast, Pink et al., using seven-day food records in 46 patients with post-COVID-19 fatigue and 46 controls, found no clear differences in energy, protein, or carbohydrate intake that explained fatigue [6]. This finding is consistent with the lack of consistent associations with MFIS in the present study, although the questions are not equivalent: comparing patients with controls does not estimate the association between intake and severity within an already affected population. Together, the two studies argue against explaining fatigue solely in terms of macronutrient quantities, while not excluding individual nutritional problems. The CUME study provides a relevant comparison. Among 2065 Brazilian adults, the highest quartiles of protein and lipid intake were associated with a higher subsequent occurrence of post-COVID-19 syndrome, with hazard ratios of 1.36 and 1.23, respectively [7]. This direction differs from the inverse association between absolute fat intake and frailty observed here. However, CUME examined the subsequent onset or presence of the syndrome, used dietary information from different baseline waves, and assessed exposures expressed as energy proportions. Our analysis examines clinical manifestations in individuals with established long COVID-19. These differences in temporality, measurement, and outcomes preclude interpreting the findings as directly contradictory or inferring that increasing or decreasing a macronutrient has therapeutic effects.

More recent studies also highlight diet quality and food sources. In 2026, Freitas et al. described associations between poorer diet quality and greater symptom burden, particularly in women, although they did not observe overall between-group differences in many dietary and biological indicators [8]. Outside the specific context of long COVID-19, a cohort of 44,465 UK Biobank participants linked greater adherence to the EAT-Lancet dietary pattern to a lower incidence of frailty during follow-up [36]. This finding supports investigating whole dietary patterns but does not validate a protective effect of total or saturated fat: it uses a different definition of frailty and an exposure integrating multiple foods. Our nutrient-based analysis cannot adequately distinguish the contribution of food sources or the food matrix. The relationship with frailty is clinically plausible but is not nutritionally specific. Appelman et al. identified muscular and metabolic abnormalities in patients with long COVID-19 and post-exertional malaise [2]. These findings show that functional limitation may involve processes other than low intake. Conversely, greater clinical impairment could make eating more difficult and contribute to the observed inverse association. The FRAIL scale includes fatigue and weight loss, dimensions that may overlap with long COVID-19 manifestations. A lower FRAIL score should therefore not be interpreted as demonstrating better muscle metabolism or the absence of sarcopenia.

Fatty acid classes also differ in their immunomodulatory actions. Experimental studies indicate that the omega-3 PUFA eicosapentaenoic acid (EPA) and docosahexaenoic acid (DHA) can suppress macrophage inflammatory signaling through G protein-coupled receptor 120 (GPR120) [37]. For MUFA, oleic acid has been shown to inhibit SFA-induced activation of the NOD-, LRR- and pyrin domain-containing protein 3 (NLRP3) inflammasome and interleukin-1β (IL-1β) release in human monocytes/macrophages. Conversely, palmitate and stearate can promote NLRP3-dependent IL-1β release under experimental conditions [38]. These findings provide biological context but do not establish that habitual dietary intake produces equivalent effects in adults with long COVID-19. Our analysis assessed total PUFA intake rather than EPA and DHA separately and did not test these immune pathways. Furthermore, the inverse association between absolute SFA intake and frailty should not be interpreted as evidence of an anti-inflammatory or protective effect of SFA.

Regarding cognition, Greene et al. described blood–brain barrier abnormalities and persistent inflammation in individuals with long COVID-19 and cognitive difficulties [39]. This context supports investigating metabolic and nutritional pathways but does not demonstrate that the exploratory MoCA curves observed in our study reflect these mechanisms. Furthermore, a 2025 trial with 58 participants found that nicotinamide riboside increased concentrations of nicotinamide adenine dinucleotide (NAD^+^) without significant between-group differences in the primary cognitive outcomes [40]. This finding illustrates that changing a bioenergetics-related marker does not guarantee clinical improvement. In our study, nominal linear associations of MoCA with carbohydrates and total fat did not survive correction for multiple testing and do not justify recommending a low-carbohydrate or high-fat diet.

Supplement trials provide complementary information but are not equivalent to studying habitual diet. Tosato et al. reported functional improvements with L-arginine plus vitamin C in a short trial [11]. In 2026, a pilot creatine trial randomized 67 individuals; 58 completed four weeks, and the 6-g/day group showed improvements in fatigue and handgrip strength relative to the control group, with concurrent physical activity in all groups [9]. By contrast, the omega-3 pilot study by Sarkar et al., in which 18 participants completed follow-up, detected no significant improvements in the symptoms assessed [10]. The small samples, duration, and specificity of these interventions limit extrapolation to total protein intake or dietary polyunsaturated fatty acids.

The microbiota represents another area of investigation. The RECOVERY trial, involving 463 participants, found greater alleviation of fatigue, memory complaints, concentration difficulties, and gastrointestinal symptoms with the synbiotic preparation SIM01 [41]. More recently, the DELong#3 trial, published in 2026, reported improvements in fatigue with a probiotic formulation versus placebo; 48 participants were analyzed after four weeks [42]. Its small sample and use of a one-sided test for change in fatigue warrant caution. These findings require confirmation and assessment of generalizability. Neither intervention allows the results to be attributed to habitual fiber intake: a synbiotic combines components, and a probiotic does not represent an isolated dietary fiber intervention. Thus, the absence of consistent associations with fiber in BioICOPER does not exclude a role for the microbiota.

Regarding comprehensive dietary interventions, ReDIRECT randomized 234 adults with long COVID-19 and overweight and observed a modest improvement in each participant’s selected primary symptom through a structured weight management program [12]. The trial was open-label and wait-list-controlled and did not isolate macronutrient effects. A 2026 feasibility trial combined nutritional counseling and physiotherapy in 65 participants; confidence intervals for between-group differences in changes in the one-minute sit-to-stand test included the null value [13]. These studies support investigating individualized interventions without establishing a universal dietary composition or justifying energy restriction for all patients.

### 4.3. Clinical Implications

The main implication is to integrate nutritional assessment into the clinical evaluation of long COVID-19, considering weight changes, appetite, gastrointestinal symptoms, alterations in taste and smell, the ability to obtain and prepare food, and actual intake. Assessment should include functional status and, when indicated, measures of strength and body composition. Overweight does not exclude nutritional risk, as shown by the clinical cohort of Slotegraaf et al. [5]. Nevertheless, our data do not demonstrate that recorded energy intake was insufficient, because it was not compared with individual requirements, nor do they allow malnutrition to be diagnosed from macronutrient intake.

The inverse association between saturated fat and frailty does not justify increasing its intake. General recommendations on diet quality and replacing saturated fats with unsaturated fats and fiber-rich foods should be maintained, in accordance with WHO guidance [23]. Likewise, exceeding the population-level protein interval of 10–15% of energy used for descriptive classification does not imply harmful protein excess. Individual adequacy requires assessing intake relative to body weight, requirements, and clinical status. The exploratory MoCA findings likewise do not support prescribing specific proportions of protein or polyunsaturated fat. Management should be tailored to the identified problem: an individual with involuntary weight loss and eating difficulties may need support to meet requirements, whereas someone with excess adiposity may benefit from a supervised program that preserves muscle function. Evidence from ReDIRECT is limited to individuals with overweight and does not support indiscriminate hypocaloric diets [12]. Recommendations should be feasible in the presence of fatigue and functional limitation, and supplements should be considered for specific indications, without extrapolating benefits from small trials to all patients.

### 4.4. Strengths and Limitations

Strengths include the sample size, dietary assessment over seven consecutive days using a standardized application, and characterization of twelve clinical outcomes covering symptoms, mental health, quality of life, frailty, dyspnea, and cognition. Distinguishing absolute intake from energy-adjusted composition facilitates more precise interpretation of the exposures. HC3 robust standard errors in linear models and Benjamini–Hochberg correction within the specified test families reduce certain inferential problems. Distinguishing subjective memory complaints from MoCA assessment also addresses complementary cognitive dimensions.

The main limitation is the cross-sectional design, which precludes establishing temporal precedence and causality. Greater clinical impairment may alter diet, in addition to the potential influence of diet on health. Models were adjusted for a limited set of covariates, leaving potential confounding by adiposity, physical activity, education, socioeconomic status, comorbidities, medications, supplements, and infection characteristics. Although MoCA scores incorporated the education-related correction, educational attainment may still influence cognitive performance and was not included as a model covariate. Future studies should consider these factors within a prespecified causal framework, avoiding indiscriminate adjustment that includes potential mediators. Dietary records rely on self-report and may be affected by portion-size estimation errors, underreporting, or behavioral changes during observation. Although all data were collected within a two-week period, the seven-day dietary records and the retrospective symptom assessments did not cover identical reference periods. Seven days provide detailed information about a specific period but may not represent long-term habitual intake or preinfection diet. Nutrient quantification also does not adequately identify food sources, degree of processing, or whole dietary patterns. Moreover, energy-percentage models adjusted for total energy do not identify a specific isocaloric substitution between nutrients, because this contrast was not explicitly defined. Recruitment from a single geographical area, the predominance of women, and the requirement to attend the assessment limit generalizability, particularly to patients with greater dependency. Excluding individuals without complete dietary records may introduce selection bias, and available-case analysis for each outcome may add bias if missingness is related to exposure or health. The absence of a recovered or uninfected comparison group prevents determining whether the observed dietary pattern is specific to long COVID-19. Several clinical outcomes were assessed using self-reported questionnaires and therefore relied on participants’ recall, perceptions, and willingness to report symptoms accurately. These measures are susceptible to recall bias, reporting bias, and subjective interpretation, and questionnaire responses could not be independently verified. Although validated instruments were used, these limitations may have introduced measurement error and influenced the observed associations. Outcome and modeling limitations should also be considered. FRAIL is not an objective measure of muscle mass or function and shares components with the symptoms studied. Collapsing the higher FRAIL and mMRC categories reduces clinical detail, and estimates may be unstable when few participants fall into a category. Interpretation of ordinal and multinomial models depends on the adequacy of their assumptions and estimation stability. HC3 standard errors do not correct functional misspecification, residual confounding, or measurement error. The RESET used was a classical F test, sensitive to its own assumptions; a nonsignificant result neither demonstrates correct functional form nor excludes specific nonlinear relationships. Finally, the large number of exposures, outcomes, and exploratory analyses warrants caution. Family-specific correction does not necessarily control multiplicity across all analytical decisions. MoCA quadratic terms were evaluated as sensitivity analyses after examining functional form; their results therefore require external validation and do not define therapeutic cutoffs. Sex-stratified correlations likewise do not demonstrate effect modification without a formal interaction test. Lack of significance after correction does not demonstrate equivalence or exclude smaller associations, whose detection may require larger samples.

## 5. Conclusions

In adults with long COVID-19 from the BioICOPER cohort, higher absolute intakes of total fat and saturated and monounsaturated fatty acids were associated with lower odds of being in a higher frailty category after adjustment for age and sex and correction for multiple testing. In sensitivity analyses additionally adjusted for total energy intake, only total fat retained a borderline nominal association with FRAIL. However, the primary analyses of energy-adjusted dietary composition showed no associations that remained significant after correction. These findings do not establish a protective effect of fats or support recommending a specific macronutrient distribution to improve clinical manifestations. The exploratory nonlinear relationships between protein, polyunsaturated fats, and MoCA require replication. Longitudinal studies with repeated dietary assessment, functional characterization, and adequate confounding control, together with trials explicitly defining the nutritional intervention, are needed to establish their potential clinical utility.

## Figures and Tables

**Table 1:** Sociodemographic and clinical characteristics of participants with long COVID-19, overall and by sex.

Characteristic	Overall (*n* = 290)	Women (*n* = 201, 69.3%)	Men (*n* = 89, 30.7%)	*p* Value
Participant characteristics				
Age, years	53.2 ± 11.8	51.7 ± 11.4	56.7 ± 12.0	<0.001
Body mass index, kg/m^2^	27.6 [23.6–31.2]	26.4 [22.7–30.7]	29.4 [26.0–32.6]	<0.001
Time since COVID-19, months	40.50 [34.98–46.45]	40.1 [35.5–45.0]	40.8 [33.7–47.0]	0.517
Hospitalization during acute COVID-19	81 (27.9%)	42 (20.9%)	39 (43.8%)	<0.001
Mediterranean diet adherence score	8 [7–9]	8 [7–9]	8 [6–9]	0.443
Alcohol intake, g/week	0 [0–32.5]	0 [0–20]	25 [0–97.5]	<0.001
Clinical variables				
Global symptom domain count	4 [2.8–5]	4 [3–6]	3 [2–5]	<0.001
Pittsburgh Sleep Quality Index total score	10 [7–13]	10 [8–14]	8 [6–12]	<0.001
Modified Fatigue Impact Scale total score	45 [20–62]	48.5 [26.8–67]	32 [13.5–53]	<0.001
MFE-30 total score	31 [16–53]	36 [18–55]	25 [13.5–42]	0.002
SF-36 physical component summary (PCS)	40.0 [31.8–48.8]	38.4 [31.4–46.6]	45.5 [34.3–50.8]	0.001
SF-36 mental component summary (MCS)	45.3 [30.6–53.4]	43.7 [29.0–52.5]	46.8 [34.3–53.9]	0.027
GADS anxiety score	4 [1–7]	5 [1–7]	1 [0–6]	<0.001
GADS depression score	4 [1–6]	4 [2–6]	2 [0–5]	<0.001
Pain score	28 [16–46]	32 [20–48]	19.5 [9.3–36.8]	<0.001
FRAIL total score	1 [0–2]	1 [0–2]	0 [0–1]	<0.001
mMRC dyspnea scale	1 [1–1]	1 [1–1]	1 [0–1]	0.046
MoCA total score	24 [21–26]	24 [22–26]	23 [21–25]	0.102

Values are mean ± standard deviation or median [25th–75th percentiles], as appropriate; categorical variables are n (%). *p* values compare women and men using the Mann–Whitney U test for continuous or ordinal variables and Pearson’s chi-square test for the hospitalization variable. *p* values are nominal and were not adjusted for multiple comparisons. Available sample sizes varied for clinical outcomes because of missing data (overall n = 284–290; women n = 196–201; men n = 87–89). FRAIL, fatigue, resistance, ambulation, illnesses, and loss of weight; GADS, Goldberg Anxiety and Depression Scale; MCS, mental component summary; MFE-30, 30-item Memory Failures of Everyday questionnaire; MFIS, Modified Fatigue Impact Scale; mMRC, modified Medical Research Council dyspnea scale; MoCA, Montreal Cognitive Assessment; PCS, physical component summary; PSQI, Pittsburgh Sleep Quality Index; SF-36, 36-Item Short Form Health Survey. The global symptom domain count ranges from 0 to 6.

**Table 2:** Daily energy and nutrient intake, macronutrient energy percentages, and fiber density, overall and by sex.

Daily Energy and Nutrient Intake	Overall (n = 290)	Women (n = 201)	Men (n = 89)	*p* Value
Energy intake, kcal/day	1717 [1384–2046]	1682 [1377–2000]	1801 [1420–2127]	0.081
Carbohydrates (g/day)	161.88 [118.35–197.62]	158.60 [117.87–196.95]	172.12 [126.67–207.44]	0.309
Protein (g/day)	76.91 [66.05–92.58]	75.36 [65.90–89.80]	80.81 [66.00–100.39]	0.107
Fiber (g/day)	21.01 [16.71–26.28]	21.04 [16.93–25.79]	20.97 [16.09–28.63]	0.828
Total fat (g/day)	79.35 [64.51–95.51]	77.38 [63.96–94.83]	81.18 [66.27–104.74]	0.127
Saturated fatty acids (g/day)	24.38 [19.59–31.03]	23.59 [19.41–29.25]	26.36 [19.79–34.31]	0.035
Monounsaturated fatty acids (g/day)	37.39 [30.99–45.61]	37.07 [30.76–43.82]	38.27 [31.74–48.26]	0.173
Polyunsaturated fatty acids (g/day)	10.11 [8.17–12.95]	10.21 [8.18–13.01]	9.90 [7.91–12.94]	0.556
Dietary composition				
Carbohydrates, % of energy	37.1 [32.8–41.3]	37.1 [33.0–41.3]	37.1 [31.9–41.9]	0.595
Protein, % of energy	18.3 [16.8–19.9]	18.3 [17.0–19.9]	18.1 [16.3–19.9]	0.776
Fiber, g/1000 kcal	12.2 [10.5–14.4]	12.3 [10.6–14.5]	12.0 [10.1–14.2]	0.236
Fat, % of energy	42.2 ± 5.2	42.3 ± 4.9	42.0 ± 5.8	0.680
Saturated fatty acids, % of energy	13.2 ± 2.2	13.1 ± 2.2	13.4 ± 2.2	0.221
Monounsaturated fatty acids, % of energy	20.1 ± 3.1	20.1 ± 2.9	19.9 ± 3.5	0.547
Polyunsaturated fatty acids, % of energy	5.4 [4.8–6.1]	5.6 [4.9–6.3]	5.0 [4.6–5.7]	<0.001

Values are mean ± standard deviation for the energy percentages from total fat, saturated fatty acids, and monounsaturated fatty acids, and median [25th–75th percentiles] for the remaining variables. All dietary variables were available for 290 participants. *p* values compare women and men using the Mann–Whitney U test and are nominal, without adjustment for multiple comparisons. Energy percentages and fiber density are descriptive measures of dietary composition; they are not regression-adjusted estimates.

## Data Availability

The BioICOPER dataset is deposited in Zenodo (https://doi.org/10.5281/zenodo.14282873). The repository record is publicly accessible, whereas access to the data files is restricted.

## Supplementary Materials

The following supporting information can be downloaded at: https://www.mdpi.com/article/10.3390/nu18213716/s1, Table S1. Dietary targets and operational definitions; Table S2. Descriptive statistics and normality assessment of study variables; Table S3. Participant characteristics by completeness of the dietary record; Table S4. Attainment of selected dietary targets overall and by sex; Figure S1. Sex-stratified Spearman correlations between daily dietary intake and clinical outcomes. Table S5. Associations between absolute dietary intake and clinical outcomes adjusted for age and sex (Model 1); Table S6. Associations between dietary macronutrient composition and clinical outcomes adjusted for age, sex, and total energy intake (Model 2); Figure S2. Nominal associations between dietary macronutrient composition and MoCA total score in Model 2; Figure S3. Exploratory quadratic associations of protein and polyunsaturated fatty acid intake with cognitive performance; Table S7. Exploratory quadratic associations between dietary composition and MoCA; Table S8. Sensitivity analyses of associations between absolute fat intake and FRAIL categories, additionally adjusted for total energy intake.

## Abbreviations

BH	Benjamini–Hochberg
CI	Confidence interval
E%	Percentage of total energy intake
FRAIL	Fatigue, Resistance, Ambulation, Illnesses, and Loss of weight
GADS	Goldberg Anxiety and Depression Scale
HC3	Heteroscedasticity-consistent covariance estimator, type 3
MCS	Mental Component Summary
MFE-30	30-item version of the Memory Failures of Everyday Questionnaire
MFIS	Modified Fatigue Impact Scale
mMRC	Modified Medical Research Council
MoCA	Montreal Cognitive Assessment
MUFA	Monounsaturated fatty acids
NAD^+^	Nicotinamide adenine dinucleotide, oxidized form
OLS	Ordinary least squares
OR	Odds ratio
PCS	Physical Component Summary
PSQI	Pittsburgh Sleep Quality Index
PUFA	Polyunsaturated fatty acids
RESET	Regression Equation Specification Error Test
SARS-CoV-2	Severe acute respiratory syndrome coronavirus 2
SD	Standard deviation
SF-36	36-Item Short Form Health Survey
SFA	Saturated fatty acids
STROBE	Strengthening the Reporting of Observational Studies in Epidemiology
APISAL	Primary Care Research Unit of Salamanca
CEIm	Research Ethics Committee for Medicines
FAO	Food and Agriculture Organization of the United Nations
IBSAL	Institute of Biomedical Research of Salamanca
ISCIII	Instituto de Salud Carlos III
REDIAPP	Research Network on Preventive Activities and Health Promotion
RICAPPS	Research Network on Chronicity, Primary Care and Health Promotion
WHO	World Health Organization
BMI	Body mass index
DHA	Docosahexaenoic acid
EPA	Eicosapentaenoic acid
GPR120	G protein-coupled receptor 120
IL-1β	Interleukin-1β
NLRP3	NOD-, LRR- and pyrin domain-containing protein 3
