# Supplementary material for: Macronutrient Intake and Clinical Manifestations in Adults with Long COVID-19: A Cross-Sectional Analysis of the BioICOPER Study
Source: Nutrients. 2026 Nov 9;18(21):3716. doi: 10.3390/nu18213716 (PMC13765583; doi:10.3390/nu18213716)
Supplement: Supplementary file 1 [file nutrients-18-03716-s001.zip › nutrients-4674673-supplementary.pdf]

Supplementary Materials

Table S1. Dietary targets and operational definitions

Dietary component	Selected target	Attainment rule	Source
Protein	10–15% energy	$10 \leq E\% \leq 15$	WHO/FAO 2003 [1]
Carbohydrate	45–60% energy	$45 \leq E\% \leq 60$	EFSA 2010 [2]
Total fat	20–35% energy	$20 \leq E\% \leq 35$	EFSA 2010 [3]
Saturated fatty acids	<10% energy	Below 10 E%	WHO 2023 [4]
Dietary fiber	≥25 g/day	At least 25 g/day	EFSA 2010 [2]

Selected population reference targets used to describe dietary intake in adults with long COVID. Energy percentages were calculated from the mean daily intake recorded over seven days: E% = 100 × intake (g/day) × conversion factor (kcal/g) / total reported energy intake (kcal/day). Conversion factors were 4 kcal/g for protein and carbohydrate and 9 kcal/g for fat. Range boundaries were included; the saturated fat criterion was strictly <10 E%. Each target was evaluated separately. These operational criteria do not constitute a validated composite diet-quality index or diagnostic criteria for individual nutritional deficiency or excess. The protein range is a population distribution goal; an intake >15 E% does not establish excessive or harmful protein intake. Individual protein adequacy in g/kg/day was not assessed. Target attainment was not classified for total MUFA or PUFA because EFSA does not specify reference intake ranges for these totals [3]. Abbreviations: E%, percentage of total reported energy intake; EFSA, European Food Safety Authority; FAO, Food and Agriculture Organization of the United Nations; MUFA, monounsaturated fatty acids; PUFA, polyunsaturated fatty acids; WHO, World Health Organization.

References

1. World Health Organization; Food and Agriculture Organization. Diet, nutrition and the prevention of chronic diseases. WHO Technical Report Series 916. Geneva: WHO; 2003. Table 6. https://www.fao.org/4/ac911e/ac911e07.htm

2. EFSA Panel on Dietetic Products, Nutrition, and Allergies. Scientific Opinion on Dietary Reference Values for carbohydrates and dietary fibre. EFSA Journal. 2010;8(3):1462. https://doi.org/10.2903/j.efsa.2010.1462

3. EFSA Panel on Dietetic Products, Nutrition, and Allergies. Scientific Opinion on Dietary Reference Values for fats, including saturated fatty acids, polyunsaturated fatty acids, monounsaturated fatty acids, trans fatty acids, and cholesterol. EFSA Journal. 2010;8(3):1461. https://doi.org/10.2903/j.efsa.2010.1461

4. World Health Organization. Saturated fatty acid and trans-fatty acid intake for adults and children: WHO guideline. Geneva: WHO; 2023. https://www.who.int/publications/i/item/9789240073630

Table S2. Descriptive statistics and normality assessment of study variables

Variable	N	Descriptive statistics	Shapiro–Wilk	p
Age, years	290	53.21 ± 11.79	0.991	0.071
Sex: women	290	201 (69.3%)	—	Not applicable
Sex: men	290	89 (30.7%)	—	Not applicable
BMI, kg/m²	290	27.61 [23.63–31.25]	0.970	<0.001
Time since infection, months	290	40.50 [34.98–46.45]	0.915	<0.001
Acute COVID-19 hospitalization: no	290	209 (72.1%)	—	Not applicable
Acute COVID-19 hospitalization: yes	290	81 (27.9%)	—	Not applicable
Mediterranean diet score	290	8 [7–9]	0.965	<0.001
Energy intake, kcal/day	290	1,716.56 [1,384.11–2,046.40]	0.967	<0.001
Alcohol, g/week	290	0 [0–32.5]	0.618	<0.001
Clinical variables				
Global symptom domain count	290	4 [2.75–5]	0.908	<0.001
PSQI total score	288	10 [7–13]	0.979	<0.001
MFIS total fatigue score	287	45 [20–62]	0.953	<0.001
MFE-30 total score	288	31 [16–53]	0.957	<0.001
SF-36 PCS	287	39.97 [31.84–48.79]	0.981	<0.001
SF-36 MCS	287	45.29 [30.58–53.44]	0.946	<0.001
GADS anxiety score	290	4 [1–7]	0.860	<0.001
GADS depression score	290	4 [1–6]	0.916	<0.001
Pain score	285	28 [16–46]	0.965	<0.001
FRAIL total score	290	1 [0–2]	0.840	<0.001
mMRC dyspnea grade	284	1 [1–1]	0.821	<0.001
MoCA total score	286	24 [21–26]	0.962	<0.001
Dietary variables				
Protein, % of energy	290	18.27 [16.82–19.93]	0.990	0.040
Carbohydrate, % of energy	290	37.11 [32.81–41.35]	0.981	<0.001
Total fat, % of energy	290	42.19 ± 5.21	0.997	0.919
SFA, % of energy	290	13.18 ± 2.20	0.992	0.119
MUFA, % of energy	290	20.07 ± 3.09	0.991	0.075
Polyunsaturated fatty acids, % of energy	290	5.37 [4.76–6.07]	0.779	<0.001
Fiber, g/1,000 kcal	290	12.24 [10.48–14.42]	0.989	0.022

The analytical sample comprised 290 participants with complete seven-day dietary records. N indicates the number of participants with available data for each variable; lower values reflect missing clinical outcome data. Quantitative variables are summarized as median [25th–75th percentiles] when the Shapiro–Wilk test indicated a departure from normality ($p < 0.05$), and as mean ± SD otherwise. Categorical variables are presented as n (%); normality testing is not applicable. W is the Shapiro–Wilk test statistic. Age and the energy percentages from total fat, SFA, and MUFA did not show statistically significant departures from normality. The global symptom domain count indicates the number of six domains with at least one persistent symptom (range, 0–6); it is not a general health rating. Normality tests alone do not determine the appropriate regression model. Abbreviations: BMI, body mass index; FRAIL, fatigue, resistance, ambulation, illnesses, and loss of weight; GADS, Goldberg Anxiety and Depression Scale; MCS, mental component summary; MFE-30, 30-item Memory Failures of Everyday questionnaire; MFIS, Modified Fatigue Impact Scale; mMRC, modified Medical Research Council dyspnea scale; MoCA, Montreal Cognitive Assessment; PCS, physical component summary; PSQI, Pittsburgh Sleep Quality Index; SF-36, 36-Item Short Form Health Survey; MUFA, monounsaturated fatty acids; SFA, saturated fatty acids; SD, standard deviation.

Table S3. Participant characteristics by completeness of the dietary record

Variable	Incomplete dietary record (n = 14)	Complete dietary record (n = 290)	p
Participant characteristics			
Age (years)	44.5 [36.5–49.5] (n=14)	53 [46–62] (n=290)	0.003
Sex, women	6 (42.9%)	201 (69.3%)	0.073†
Sex, men	8 (57.1%)	89 (30.7%)	
BMI (kg/m²)	27.5 [22.2–30.8] (n=14)	27.6 [23.6–31.2] (n=290)	0.663
Time since infection (months)	38.0 [30.6–43.5] (n=14)	40.50 [34.98–46.45] (n=290)	0.366
Acute COVID-19 hospitalization, yes	2 (14.3%)	81 (27.9%)	0.365†
Mediterranean diet score	6.5 [0–9] (n=14)	8 [7–9] (n=290)	0.101
Alcohol intake (g/week)	12.5 [0–31.3] (n=14)	0 [0–32.5] (n=290)	0.613
Global symptom domain count	4.5 [2–6] (n=14)	4 [2.8–5] (n=290)	0.644
Sleep quality (PSQI total)	8.5 [3.5–13.3] (n=10)	10 [7–13] (n=288)	0.163
Fatigue (MFIS total)	46 [21–67.5] (n=9)	45 [20–62] (n=287)	0.870
MFE-30 total score	34.5 [20.3–57] (n=10)	31 [16–53] (n=288)	0.775
SF-36 PCS	45.3 [33.6–53.7] (n=9)	40.0 [31.8–48.8] (n=287)	0.280
SF-36 MCS	40.0 [29.3–52.6] (n=9)	45.3 [30.6–53.4] (n=287)	0.585
Anxiety score (GADS)	4.5 [0.8–7] (n=14)	4 [1–7] (n=290)	0.736
Depression score (GADS)	4 [0–6] (n=14)	4 [1–6] (n=290)	0.995
Pain score	16 [5.3–31.5] (n=10)	28 [16–46] (n=285)	0.054
Frailty score (FRAIL)	1 [0–1] (n=14)	1 [0–2] (n=290)	0.366
Dyspnea (mMRC)	1 [0.8–1] (n=10)	1 [1–1] (n=284)	0.686
Cognitive function (MoCA total)	23 [22.5–25] (n=13)	24 [21–26] (n=286)	0.574

Continuous and ordinal variables are presented as median [25th–75th percentiles] and were compared using the Mann–Whitney U test. Categorical variables are presented as n (%) within each group; † indicates a two-sided Fisher exact test. Sex was compared as a single categorical variable, with its p value displayed on the women row. The hospitalization p value was calculated using the displayed counts (2/14 vs. 81/290). Sample sizes are shown for quantitative variables because the availability of clinical data varied. All p values are unadjusted for multiple comparisons. The global symptom domain count ranges from 0 to 6. Energy intake was not compared because complete dietary records were unavailable in the excluded group. Abbreviations: BMI, body mass index; FRAIL, fatigue, resistance, ambulation, illnesses, and loss of weight; GADS, Goldberg Anxiety and Depression Scale; MCS, mental component summary; MFE-30, 30-item Memory Failures of Everyday questionnaire; MFIS, Modified Fatigue Impact Scale; mMRC, modified Medical Research Council dyspnea scale; MoCA, Montreal Cognitive Assessment; PCS, physical component summary; PSQI, Pittsburgh Sleep Quality Index; SF-36, 36-Item Short Form Health Survey.

Table S4. Attainment of selected dietary targets overall and by sex

Dietary target	Overall<br>N = 290	Men<br>N = 89	Women<br>N = 201	p	q
Protein<br>10–15% energy	23/290<br>7.9% (5.3–11.6)	8/89<br>9.0% (4.6–16.7)	15/201<br>7.5% (4.6–11.9)	0.644	0.796
Carbohydrate<br>45–60% energy	23/290<br>7.9% (5.3–11.6)	9/89<br>10.1% (5.4–18.1)	14/201<br>7.0% (4.2–11.4)	0.355	0.796
Total fat<br>20–35% energy	25/290<br>8.6% (5.9–12.4)	9/89<br>10.1% (5.4–18.1)	16/201<br>8.0% (5.0–12.5)	0.650	0.796
Saturated fatty acids<br><10% energy	18/290<br>6.2% (4.0–9.6)	6/89<br>6.7% (3.1–13.9)	12/201<br>6.0% (3.4–10.1)	0.796	0.796
Dietary fiber<br>≥25 g/day	85/290<br>29.3% (24.4–34.8)	30/89<br>33.7% (24.7–44.0)	55/201<br>27.4% (21.7–33.9)	0.328	0.796

The analytical sample comprised 290 adults with long COVID and complete seven-day dietary records. Values are the number meeting the target/number assessed, followed by the percentage (95% CI). Confidence intervals were calculated using the Wilson score method. Two-sided Fisher exact tests compared men and women; q values were calculated using the Benjamini–Hochberg procedure across these five comparisons. The overall sample includes both sexes and was not treated as an independent comparison group. Attainment refers to the selected population dietary targets defined in Table S1 and does not establish individual nutritional adequacy. Abbreviations: CI, confidence interval; N, number of participants.

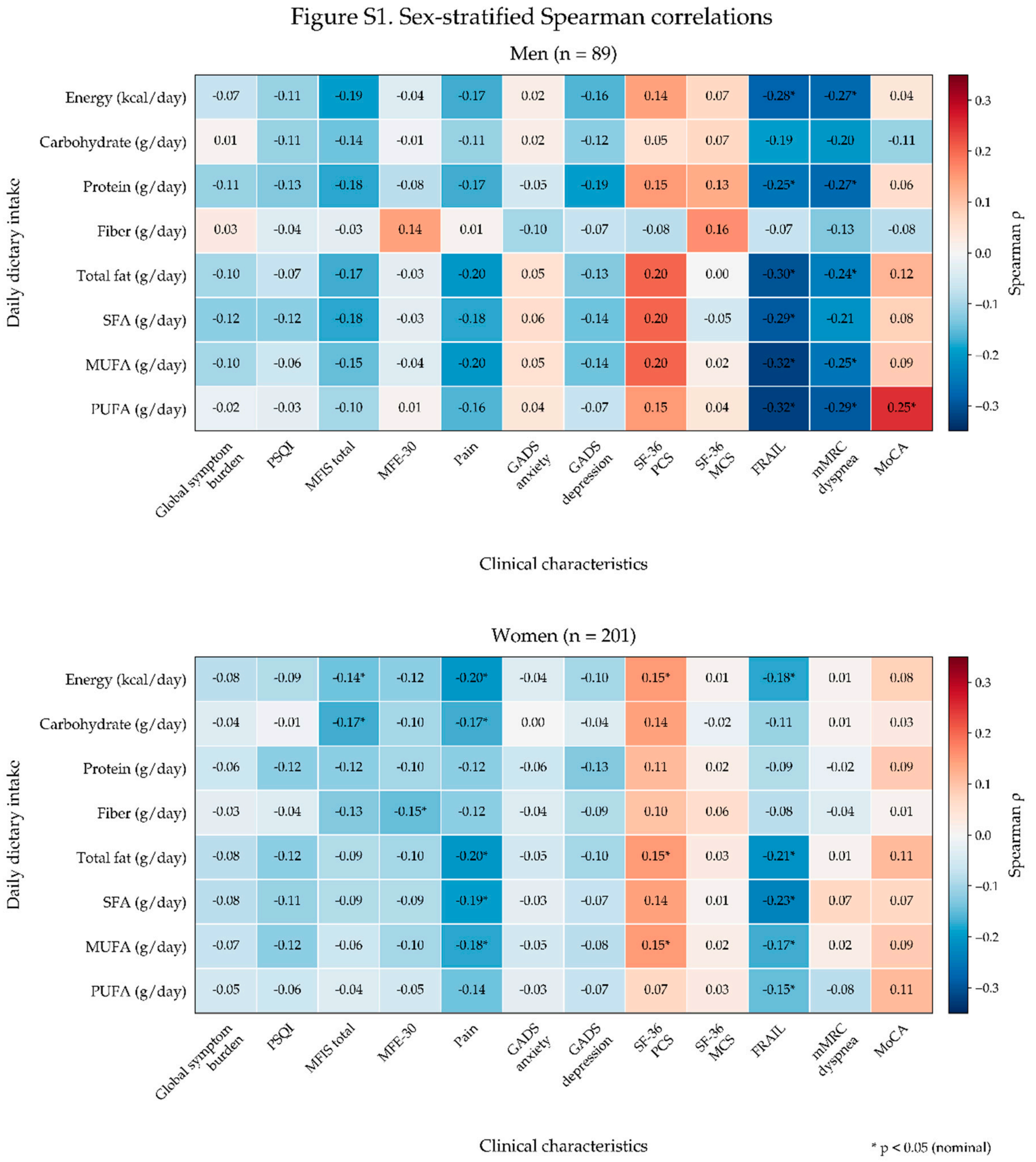

Figure S1. Sex-stratified Spearman correlations between daily dietary intake and clinical outcomes. Cells show Spearman correlation coefficients (ϱ) for men (n = 89) and women (n = 201), using available pairs of observations. Blue indicates negative correlations and red indicates positive correlations. * Nominal $p < 0.05$; no correction for multiple comparisons was applied. The figure is exploratory and does not establish that associations differ between sexes. Global symptom burden represents the number of six symptom domains with at least one persistent manifestation (range, 0–6). Abbreviations: FRAIL, fatigue, resistance, ambulation, illnesses, and loss of weight; GADS, Goldberg Anxiety and Depression Scale; MCS, mental component summary; MFE-30, 30-item Memory Failures of Everyday questionnaire; MFIS, Modified Fatigue Impact Scale; mMRC, modified Medical Research Council dyspnea scale; MoCA, Montreal Cognitive Assessment; MUFA, monounsaturated fatty acids; PCS, physical component summary; PSQI, Pittsburgh Sleep Quality Index; PUFA, polyunsaturated fatty acids; SFA, saturated fatty acids; SF-36, 36-Item Short Form Health Survey.

Table S5. Associations between absolute dietary intake and clinical outcomes adjusted for age and sex (Model 1)

Clinical outcome	Dietary exposure	N	Estimate (95% CI) or global test	p	q (BH)
Global symptom domain count	Energy intake (kcal/day)	290	B = -0.000130 (-0.000569, 0.000309)	0.561	0.719
Global symptom domain count	Protein (g/day)	290	B = -0.004 (-0.014, 0.007)	0.491	0.664
Global symptom domain count	Fiber (g/day)	290	B = -0.001 (-0.032, 0.030)	0.941	0.961
Global symptom domain count	Total fat (g/day)	290	B = -0.004 (-0.013, 0.005)	0.345	0.562
Global symptom domain count	SFA (g/day)	290	B = -0.012 (-0.037, 0.013)	0.330	0.562
Global symptom domain count	MUFA (g/day)	290	B = -0.008 (-0.027, 0.012)	0.429	0.614
Global symptom domain count	PUFA (g/day)	290	B = -0.019 (-0.059, 0.021)	0.343	0.562
Global symptom domain count	Carbohydrate (g/day)	290	B = 0.000033 (-0.003563, 0.003629)	0.985	0.985
PSQI total score	Energy intake (kcal/day)	288	B = -0.001 (-0.002, 0.001)	0.341	0.562
PSQI total score	Protein (g/day)	288	B = -0.018 (-0.047, 0.010)	0.210	0.515
PSQI total score	Fiber (g/day)	288	B = -0.017 (-0.091, 0.057)	0.658	0.789
PSQI total score	Total fat (g/day)	288	B = -0.015 (-0.042, 0.011)	0.263	0.549
PSQI total score	SFA (g/day)	288	B = -0.041 (-0.113, 0.031)	0.261	0.549
PSQI total score	MUFA (g/day)	288	B = -0.028 (-0.088, 0.033)	0.370	0.574
PSQI total score	PUFA (g/day)	288	B = -0.064 (-0.161, 0.034)	0.202	0.515
PSQI total score	Carbohydrate (g/day)	288	B = -0.001 (-0.010, 0.007)	0.740	0.856
MFIS total score	Energy intake (kcal/day)	287	B = -0.006 (-0.012, 0.001)	0.076	0.374
MFIS total score	Protein (g/day)	287	B = -0.133 (-0.277, 0.010)	0.069	0.374
MFIS total score	Fiber (g/day)	287	B = -0.191 (-0.576, 0.195)	0.332	0.562
MFIS total score	Total fat (g/day)	287	B = -0.084 (-0.217, 0.049)	0.217	0.515
MFIS total score	SFA (g/day)	287	B = -0.237 (-0.611, 0.137)	0.213	0.515
MFIS total score	MUFA (g/day)	287	B = -0.130 (-0.416, 0.155)	0.371	0.574
MFIS total score	PUFA (g/day)	287	B = -0.360 (-0.865, 0.144)	0.162	0.480
MFIS total score	Carbohydrate (g/day)	287	B = -0.043 (-0.089, 0.004)	0.075	0.374
MFE-30 total score	Energy intake (kcal/day)	288	B = -0.004 (-0.010, 0.002)	0.176	0.480
MFE-30 total score	Protein (g/day)	288	B = -0.111 (-0.258, 0.035)	0.137	0.480
MFE-30 total score	Fiber (g/day)	288	B = -0.204 (-0.587, 0.178)	0.295	0.562
MFE-30 total score	Total fat (g/day)	288	B = -0.069 (-0.198, 0.060)	0.294	0.562
MFE-30 total score	SFA (g/day)	288	B = -0.150 (-0.505, 0.206)	0.409	0.602
MFE-30 total score	MUFA (g/day)	288	B = -0.161 (-0.447, 0.125)	0.270	0.552
MFE-30 total score	PUFA (g/day)	288	B = -0.196 (-0.699, 0.307)	0.445	0.619
MFE-30 total score	Carbohydrate (g/day)	288	B = -0.028 (-0.074, 0.018)	0.231	0.528
SF-36 PCS	Energy intake (kcal/day)	287	B = 0.002 (-0.001, 0.005)	0.107	0.469

Clinical outcome	Dietary exposure	N	Estimate (95% CI) or global test	p	q (BH)
SF-36 PCS	Protein (g/day)	287	B = 0.041 (-0.025, 0.107)	0.220	0.515
SF-36 PCS	Fiber (g/day)	287	B = 0.052 (-0.127, 0.232)	0.569	0.719
SF-36 PCS	Total fat (g/day)	287	B = 0.055 (-0.010, 0.120)	0.098	0.446
SF-36 PCS	SFA (g/day)	287	B = 0.133 (-0.039, 0.305)	0.130	0.480
SF-36 PCS	MUFA (g/day)	287	B = 0.104 (-0.044, 0.252)	0.169	0.480
SF-36 PCS	PUFA (g/day)	287	B = 0.264 (0.025, 0.504)	0.031*	0.268
SF-36 PCS	Carbohydrate (g/day)	287	B = 0.012 (-0.008, 0.032)	0.246	0.549
SF-36 MCS	Energy intake (kcal/day)	287	B = 0.000367 (-0.002904, 0.003639)	0.826	0.901
SF-36 MCS	Protein (g/day)	287	B = 0.024 (-0.055, 0.103)	0.548	0.719
SF-36 MCS	Fiber (g/day)	287	B = 0.108 (-0.116, 0.332)	0.345	0.562
SF-36 MCS	Total fat (g/day)	287	B = 0.011 (-0.054, 0.077)	0.733	0.856
SF-36 MCS	SFA (g/day)	287	B = -0.012 (-0.189, 0.164)	0.891	0.959
SF-36 MCS	MUFA (g/day)	287	B = 0.028 (-0.119, 0.175)	0.709	0.841
SF-36 MCS	PUFA (g/day)	287	B = 0.099 (-0.167, 0.365)	0.466	0.639
SF-36 MCS	Carbohydrate (g/day)	287	B = -0.003 (-0.030, 0.023)	0.807	0.901
GADS anxiety score	Energy intake (kcal/day)	290	B = 0.000243 (-0.000494, 0.000980)	0.518	0.690
GADS anxiety score	Protein (g/day)	290	B = 0.002 (-0.016, 0.020)	0.817	0.901
GADS anxiety score	Fiber (g/day)	290	B = 0.002 (-0.054, 0.059)	0.931	0.961
GADS anxiety score	Total fat (g/day)	290	B = 0.001 (-0.014, 0.015)	0.921	0.961
GADS anxiety score	SFA (g/day)	290	B = 0.006 (-0.034, 0.046)	0.787	0.899
GADS anxiety score	MUFA (g/day)	290	B = 0.002 (-0.031, 0.034)	0.913	0.961
GADS anxiety score	PUFA (g/day)	290	B = 0.009 (-0.068, 0.085)	0.822	0.901
GADS anxiety score	Carbohydrate (g/day)	290	B = 0.003 (-0.003, 0.009)	0.297	0.562
GADS depression score	Energy intake (kcal/day)	290	B = -0.000351 (-0.001018, 0.000316)	0.302	0.562
GADS depression score	Protein (g/day)	290	B = -0.011 (-0.027, 0.005)	0.180	0.480
GADS depression score	Fiber (g/day)	290	B = -0.019 (-0.064, 0.026)	0.402	0.602
GADS depression score	Total fat (g/day)	290	B = -0.008 (-0.022, 0.006)	0.263	0.549
GADS depression score	SFA (g/day)	290	B = -0.018 (-0.055, 0.020)	0.355	0.569
GADS depression score	MUFA (g/day)	290	B = -0.014 (-0.045, 0.018)	0.396	0.602
GADS depression score	PUFA (g/day)	290	B = -0.045 (-0.110, 0.020)	0.172	0.480
GADS depression score	Carbohydrate (g/day)	290	B = -0.001 (-0.006, 0.004)	0.641	0.780
Pain score	Energy intake (kcal/day)	285	B = -0.006 (-0.011, -0.001)	0.027*	0.257
Pain score	Protein (g/day)	285	B = -0.088 (-0.203, 0.028)	0.136	0.480
Pain score	Fiber (g/day)	285	B = -0.211 (-0.505, 0.084)	0.161	0.480
Pain score	Total fat (g/day)	285	B = -0.122 (-0.238, -0.007)	0.038*	0.301
Pain score	SFA (g/day)	285	B = -0.3007 (-0.6008, -0.0006)	0.0496*	0.340
Pain score	MUFA (g/day)	285	B = -0.240 (-0.502, 0.023)	0.073	0.374

Clinical outcome	Dietary exposure	N	Estimate (95% CI) or global test	p	q (BH)
Pain score	PUFA (g/day)	285	B = -0.532 (-0.903, -0.162)	0.005*	0.093
Pain score	Carbohydrate (g/day)	285	B = -0.034474 (-0.068832, -0.000116)	0.0492*	0.340
MoCA total score	Energy intake (kcal/day)	286	B = 0.000588 (-0.000196, 0.001372)	0.141	0.480
MoCA total score	Protein (g/day)	286	B = 0.015 (-0.004, 0.033)	0.127	0.480
MoCA total score	Fiber (g/day)	286	B = -0.003 (-0.054, 0.048)	0.900	0.959
MoCA total score	Total fat (g/day)	286	B = 0.019 (0.003, 0.035)	0.023*	0.251
MoCA total score	SFA (g/day)	286	B = 0.037 (-0.010, 0.085)	0.124	0.480
MoCA total score	MUFA (g/day)	286	B = 0.040 (0.006, 0.074)	0.021*	0.251
MoCA total score	PUFA (g/day)	286	B = 0.071 (-0.025, 0.167)	0.148	0.480
MoCA total score	Carbohydrate (g/day)	286	B = 0.000 (-0.006, 0.006)	0.959	0.969
FRAIL category (0, 1, 2, ≥3)	Energy intake (kcal/day)	290	OR = 0.999338 (0.998894, 0.999782)	0.003*	0.083
FRAIL category (0, 1, 2, ≥3)	Protein (g/day)	290	OR = 0.990 (0.980, 1.001)	0.062	0.374
FRAIL category (0, 1, 2, ≥3)	Fiber (g/day)	290	OR = 0.979 (0.950, 1.010)	0.177	0.480
FRAIL category (0, 1, 2, ≥3)	Total fat (g/day)	290	OR = 0.983 (0.974, 0.992)	<0.001*	0.034†
FRAIL category (0, 1, 2, ≥3)	SFA (g/day)	290	OR = 0.956 (0.932, 0.981)	<0.001*	0.034†
FRAIL category (0, 1, 2, ≥3)	MUFA (g/day)	290	OR = 0.967 (0.948, 0.987)	0.001*	0.038†
FRAIL category (0, 1, 2, ≥3)	PUFA (g/day)	290	OR = 0.933 (0.887, 0.982)	0.008*	0.132
FRAIL category (0, 1, 2, ≥3)	Carbohydrate (g/day)	290	OR = 0.996794 (0.993241, 1.000359)	0.078	0.374
mMRC category (0, 1, 2, ≥3)	Energy intake (kcal/day)	284	Global LR χ^2 = 2.705 (df = 3)	0.439	0.619
mMRC category (0, 1, 2, ≥3)	Protein (g/day)	284	Global LR χ^2 = 3.594 (df = 3)	0.309	0.562
mMRC category (0, 1, 2, ≥3)	Fiber (g/day)	284	Global LR χ^2 = 3.583 (df = 3)	0.310	0.562
mMRC category (0, 1, 2, ≥3)	Total fat (g/day)	284	Global LR χ^2 = 2.857 (df = 3)	0.414	0.602
mMRC category (0, 1, 2, ≥3)	SFA (g/day)	284	Global LR χ^2 = 2.029 (df = 3)	0.566	0.719
mMRC category (0, 1, 2, ≥3)	MUFA (g/day)	284	Global LR χ^2 = 1.870 (df = 3)	0.600	0.748
mMRC category (0, 1, 2, ≥3)	PUFA (g/day)	284	Global LR χ^2 = 9.485 (df = 3)	0.023*	0.251
mMRC category (0, 1, 2, ≥3)	Carbohydrate (g/day)	284	Global LR χ^2 = 1.679 (df = 3)	0.642	0.780

Each of the eight dietary exposures was evaluated separately for each of the 12 clinical outcomes in models adjusted for age and sex. Continuous outcomes were analyzed using ordinary least-squares regression with HC3 robust standard errors; estimates are B coefficients (95% CIs). FRAIL scores were grouped as 0, 1, 2, and ≥3 and analyzed using proportional-odds ordinal logistic regression; estimates are ORs (95% CIs) for being in a higher category. mMRC grades were grouped as 0, 1, 2, and ≥3 and analyzed using multinomial logistic regression, with 0 as the reference category. The global LR χ^2 test for each dietary exposure is reported with 3 df; no single OR summarizes the multinomial model. Estimates correspond to a 1-kcal/day increase in energy intake or a 1-g/day increase in each other exposure. The Benjamini–Hochberg procedure was applied across all 96 primary tests. * Nominal $p < 0.05$; † BH-adjusted $q < 0.05$. Significance markers reflect unrounded p values. Additional decimal places are retained for selected small coefficients, near-zero CI limits, and OR limits close to 1 to avoid rounding ambiguity. Fourteen associations were nominally significant, and three remained significant after BH correction. Analyses used available cases without imputation; N varies by outcome. Abbreviations: B, unstandardized regression coefficient; BH, Benjamini–Hochberg; CI, confidence interval; df, degrees of freedom; FRAIL, fatigue, resistance, ambulation, illnesses, and loss of weight; GADS, Goldberg Anxiety and Depression Scale; HC3, heteroscedasticity-consistent covariance estimator, type 3; LR, likelihood ratio; MCS, mental component summary; MFE-30, 30-item Memory Failures of Everyday questionnaire; MFIS, Modified Fatigue Impact Scale; mMRC, modified Medical Research Council dyspnea scale; MoCA, Montreal Cognitive

Assessment; MUFA, monounsaturated fatty acids; OR, odds ratio; PCS, physical component summary; PSQI, Pittsburgh Sleep Quality Index; PUFA, polyunsaturated fatty acids; SFA, saturated fatty acids; SF-36, 36-Item Short Form Health Survey.

Table S6. Associations between dietary macronutrient composition and clinical outcomes adjusted for age, sex, and total energy intake (Model 2)

Clinical outcome	Dietary exposure	N	Estimate (95% CI) or global test	p	q (BH)
Global symptom domain count	Protein (% energy)	290	B = -0.010 (-0.098, 0.079)	0.832	0.958
Global symptom domain count	Carbohydrate (% energy)	290	B = 0.020 (-0.015, 0.054)	0.260	0.861
Global symptom domain count	Total fat (% energy)	290	B = -0.021 (-0.062, 0.019)	0.304	0.861
Global symptom domain count	SFA (% energy)	290	B = -0.064 (-0.158, 0.030)	0.181	0.861
Global symptom domain count	MUFA (% energy)	290	B = -0.021 (-0.089, 0.047)	0.547	0.919
Global symptom domain count	PUFA (% energy)	290	B = -0.007 (-0.152, 0.138)	0.925	0.971
Global symptom domain count	Fiber (g/1,000 kcal)	290	B = 0.027 (-0.046, 0.099)	0.472	0.861
PSQI total score	Protein (% energy)	288	B = -0.058 (-0.305, 0.188)	0.642	0.952
PSQI total score	Carbohydrate (% energy)	288	B = 0.062 (-0.021, 0.145)	0.141	0.861
PSQI total score	Total fat (% energy)	288	B = -0.062 (-0.163, 0.039)	0.228	0.861
PSQI total score	SFA (% energy)	288	B = -0.128 (-0.366, 0.111)	0.295	0.861
PSQI total score	MUFA (% energy)	288	B = -0.079 (-0.267, 0.109)	0.412	0.861
PSQI total score	PUFA (% energy)	288	B = -0.032 (-0.363, 0.300)	0.851	0.958
PSQI total score	Fiber (g/1,000 kcal)	288	B = 0.071 (-0.112, 0.254)	0.446	0.861
MFIS total score	Protein (% energy)	287	B = 0.101 (-1.127, 1.329)	0.872	0.964
MFIS total score	Carbohydrate (% energy)	287	B = -0.069 (-0.526, 0.387)	0.766	0.952
MFIS total score	Total fat (% energy)	287	B = 0.204 (-0.359, 0.767)	0.478	0.861
MFIS total score	SFA (% energy)	287	B = 0.247 (-1.103, 1.596)	0.720	0.952
MFIS total score	MUFA (% energy)	287	B = 0.417 (-0.576, 1.409)	0.411	0.861
MFIS total score	PUFA (% energy)	287	B = 0.657 (-1.173, 2.488)	0.481	0.861
MFIS total score	Fiber (g/1,000 kcal)	287	B = 0.508 (-0.449, 1.465)	0.298	0.861
MFE-30 total score	Protein (% energy)	288	B = 0.075 (-1.220, 1.369)	0.910	0.968
MFE-30 total score	Carbohydrate (% energy)	288	B = 0.101 (-0.327, 0.528)	0.645	0.952
MFE-30 total score	Total fat (% energy)	288	B = 0.014 (-0.520, 0.548)	0.959	0.981
MFE-30 total score	SFA (% energy)	288	B = 0.234 (-1.032, 1.500)	0.717	0.952
MFE-30 total score	MUFA (% energy)	288	B = -0.217 (-1.205, 0.770)	0.666	0.952
MFE-30 total score	PUFA (% energy)	288	B = 0.756 (-0.948, 2.459)	0.384	0.861
MFE-30 total score	Fiber (g/1,000 kcal)	288	B = 0.177 (-0.805, 1.159)	0.724	0.952
SF-36 PCS	Protein (% energy)	287	B = -0.274 (-0.837, 0.288)	0.339	0.861
SF-36 PCS	Carbohydrate (% energy)	287	B = -0.087 (-0.281, 0.107)	0.379	0.861
SF-36 PCS	Total fat (% energy)	287	B = 0.149 (-0.113, 0.410)	0.265	0.861
SF-36 PCS	SFA (% energy)	287	B = 0.263 (-0.383, 0.909)	0.425	0.861
SF-36 PCS	MUFA (% energy)	287	B = 0.199 (-0.244, 0.642)	0.379	0.861

Clinical outcome	Dietary exposure	N	Estimate (95% CI) or global test	p	q (BH)
SF-36 PCS	PUFA (% energy)	287	B = 0.154 (-0.858, 1.165)	0.766	0.952
SF-36 PCS	Fiber (g/1,000 kcal)	287	B = -0.275 (-0.743, 0.193)	0.250	0.861
SF-36 MCS	Protein (% energy)	287	B = 0.239 (-0.466, 0.943)	0.506	0.886
SF-36 MCS	Carbohydrate (% energy)	287	B = -0.143 (-0.408, 0.122)	0.291	0.861
SF-36 MCS	Total fat (% energy)	287	B = 0.049 (-0.243, 0.342)	0.740	0.952
SF-36 MCS	SFA (% energy)	287	B = -0.310 (-0.987, 0.367)	0.369	0.861
SF-36 MCS	MUFA (% energy)	287	B = 0.112 (-0.430, 0.655)	0.685	0.952
SF-36 MCS	PUFA (% energy)	287	B = 0.364 (-0.516, 1.244)	0.417	0.861
SF-36 MCS	Fiber (g/1,000 kcal)	287	B = 0.277 (-0.310, 0.863)	0.355	0.861
GADS anxiety score	Protein (% energy)	290	B = -0.043 (-0.206, 0.120)	0.601	0.952
GADS anxiety score	Carbohydrate (% energy)	290	B = 0.025 (-0.034, 0.084)	0.403	0.861
GADS anxiety score	Total fat (% energy)	290	B = -0.025 (-0.093, 0.043)	0.473	0.861
GADS anxiety score	SFA (% energy)	290	B = -0.022 (-0.182, 0.137)	0.783	0.954
GADS anxiety score	MUFA (% energy)	290	B = -0.034 (-0.155, 0.088)	0.589	0.951
GADS anxiety score	PUFA (% energy)	290	B = 0.015 (-0.238, 0.268)	0.910	0.968
GADS anxiety score	Fiber (g/1,000 kcal)	290	B = -0.030 (-0.169, 0.110)	0.677	0.952
GADS depression score	Protein (% energy)	290	B = -0.054 (-0.191, 0.083)	0.440	0.861
GADS depression score	Carbohydrate (% energy)	290	B = 0.023 (-0.024, 0.070)	0.336	0.861
GADS depression score	Total fat (% energy)	290	B = -0.009 (-0.066, 0.049)	0.771	0.952
GADS depression score	SFA (% energy)	290	B = 0.003 (-0.138, 0.144)	0.970	0.981
GADS depression score	MUFA (% energy)	290	B = 0.004 (-0.100, 0.107)	0.945	0.980
GADS depression score	PUFA (% energy)	290	B = -0.024 (-0.241, 0.192)	0.825	0.958
GADS depression score	Fiber (g/1,000 kcal)	290	B = -0.011 (-0.121, 0.099)	0.842	0.958
Pain score	Protein (% energy)	285	B = 0.761 (-0.133, 1.655)	0.095	0.861
Pain score	Carbohydrate (% energy)	285	B = 0.158 (-0.149, 0.466)	0.313	0.861
Pain score	Total fat (% energy)	285	B = -0.259 (-0.653, 0.135)	0.198	0.861
Pain score	SFA (% energy)	285	B = -0.394 (-1.259, 0.472)	0.373	0.861
Pain score	MUFA (% energy)	285	B = -0.327 (-1.043, 0.389)	0.371	0.861
Pain score	PUFA (% energy)	285	B = -0.402 (-1.628, 0.825)	0.521	0.893
Pain score	Fiber (g/1,000 kcal)	285	B = 0.387 (-0.365, 1.139)	0.313	0.861
MoCA total score	Protein (% energy)	286	B = 0.000 (-0.163, 0.164)	0.996	0.996
MoCA total score	Carbohydrate (% energy)	286	B = -0.082 (-0.148, -0.016)	0.015*	0.861
MoCA total score	Total fat (% energy)	286	B = 0.079 (0.002, 0.155)	0.043*	0.861
MoCA total score	SFA (% energy)	286	B = 0.040 (-0.133, 0.213)	0.651	0.952
MoCA total score	MUFA (% energy)	286	B = 0.117 (-0.012, 0.246)	0.076	0.861
MoCA total score	PUFA (% energy)	286	B = 0.178 (-0.157, 0.514)	0.298	0.861
MoCA total score	Fiber (g/1,000 kcal)	286	B = -0.109 (-0.250, 0.032)	0.131	0.861
FRAIL category (0, 1, 2, ≥3)	Protein (% energy)	290	OR = 1.098 (0.999, 1.208)	0.054	0.861
FRAIL category (0, 1, 2, ≥3)	Carbohydrate (% energy)	290	OR = 1.020 (0.986, 1.056)	0.249	0.861

Clinical outcome	Dietary exposure	N	Estimate (95% CI) or global test	p	q (BH)
FRAIL category (0, 1, 2, ≥3)	Total fat (% energy)	290	OR = 0.965 (0.925, 1.007)	0.099	0.861
FRAIL category (0, 1, 2, ≥3)	SFA (% energy)	290	OR = 0.913 (0.828, 1.007)	0.068	0.861
FRAIL category (0, 1, 2, ≥3)	MUFA (% energy)	290	OR = 0.964 (0.896, 1.038)	0.334	0.861
FRAIL category (0, 1, 2, ≥3)	PUFA (% energy)	290	OR = 0.958 (0.824, 1.114)	0.579	0.951
FRAIL category (0, 1, 2, ≥3)	Fiber (g/1,000 kcal)	290	OR = 1.053 (0.976, 1.135)	0.183	0.861
mMRC category (0, 1, 2, ≥3)	Protein (% energy)	284	Global LR χ^2 = 1.299 (df = 3)	0.729	0.952
mMRC category (0, 1, 2, ≥3)	Carbohydrate (% energy)	284	Global LR χ^2 = 0.968 (df = 3)	0.809	0.958
mMRC category (0, 1, 2, ≥3)	Total fat (% energy)	284	Global LR χ^2 = 0.562 (df = 3)	0.905	0.968
mMRC category (0, 1, 2, ≥3)	SFA (% energy)	284	Global LR χ^2 = 4.954 (df = 3)	0.175	0.861
mMRC category (0, 1, 2, ≥3)	MUFA (% energy)	284	Global LR χ^2 = 0.775 (df = 3)	0.856	0.958
mMRC category (0, 1, 2, ≥3)	PUFA (% energy)	284	Global LR χ^2 = 7.768 (df = 3)	0.051	0.861
mMRC category (0, 1, 2, ≥3)	Fiber (g/1,000 kcal)	284	Global LR χ^2 = 1.332 (df = 3)	0.722	0.952

Each of the seven dietary exposures was evaluated separately for each of the 12 clinical outcomes in models adjusted for age, sex, and total energy intake. Continuous outcomes were analyzed using ordinary least-squares regression with HC3 robust standard errors; estimates are B coefficients (95% CIs). FRAIL scores were grouped as 0, 1, 2, and ≥3 and analyzed using proportional-odds ordinal logistic regression; estimates are ORs (95% CIs) for being in a higher category. mMRC grades were grouped as 0, 1, 2, and ≥3 and analyzed using multinomial logistic regression, with 0 as the reference category; the global LR χ^2 test is reported with 3 df. Macronutrient estimates correspond to a one-percentage-point increase in the contribution to total energy intake; fiber estimates correspond to a 1-g/1,000-kcal increase. The Benjamini–Hochberg procedure was applied across all 84 primary tests. * Nominal $p < 0.05$. Two associations were nominally significant; none had $q < 0.05$. Analyses used available cases without imputation; N varies by outcome. These are the main models with linear exposure terms; exploratory quadratic analyses of MoCA are presented separately in Figure S3 and Table S7. Abbreviations: B, unstandardized regression coefficient; BH, Benjamini–Hochberg; CI, confidence interval; df, degrees of freedom; FRAIL, fatigue, resistance, ambulation, illnesses, and loss of weight; GADS, Goldberg Anxiety and Depression Scale; HC3, heteroscedasticity-consistent covariance estimator, type 3; LR, likelihood ratio; MCS, mental component summary; MFE-30, 30-item Memory Failures of Everyday questionnaire; MFIS, Modified Fatigue Impact Scale; mMRC, modified Medical Research Council dyspnea scale; MoCA, Montreal Cognitive Assessment; MUFA, monounsaturated fatty acids; OR, odds ratio; PCS, physical component summary; PSQI, Pittsburgh Sleep Quality Index; PUFA, polyunsaturated fatty acids; SFA, saturated fatty acids; SF-36, 36-Item Short Form Health Survey.

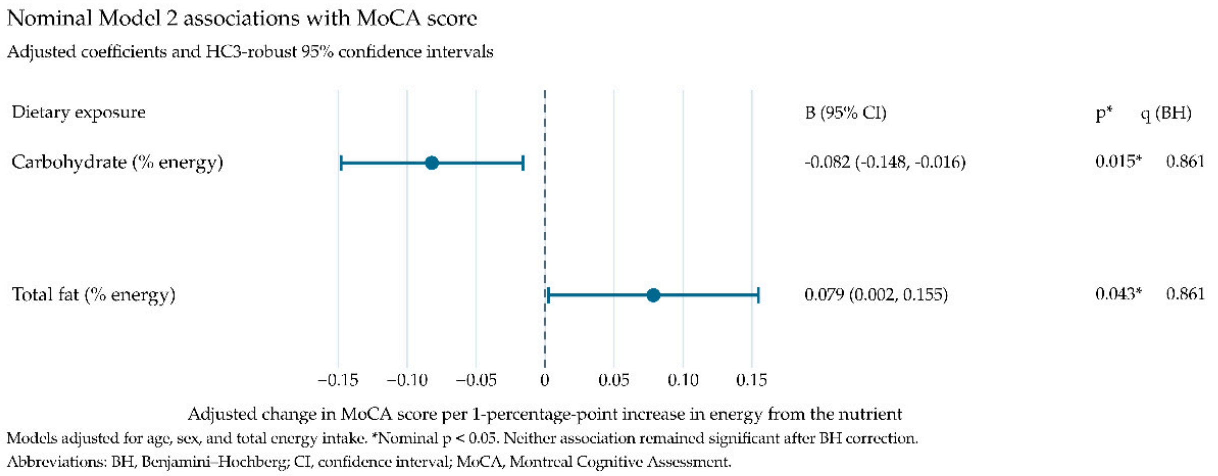

Figure S2. Nominal associations between dietary macronutrient composition and MoCA total score in Model 2. Points represent unstandardized regression coefficients (B), and horizontal lines represent 95% confidence intervals calculated using HC3 robust standard errors. Each exposure was evaluated in a separate linear model adjusted for age, sex, and total energy intake (n = 286). Coefficients indicate the estimated difference in MoCA points per one-percentage-point increase in energy from the specified nutrient. The dashed vertical line indicates no association (B = 0). Only the two associations with nominal $p < 0.05$ are shown; neither remained significant after Benjamini–Hochberg correction across the 84 primary Model 2 tests (q = 0.861 for both). Abbreviations: B, unstandardized regression coefficient; BH, Benjamini–Hochberg; CI, confidence interval; HC3, heteroscedasticity-consistent covariance estimator, type 3; MoCA, Montreal Cognitive Assessment.

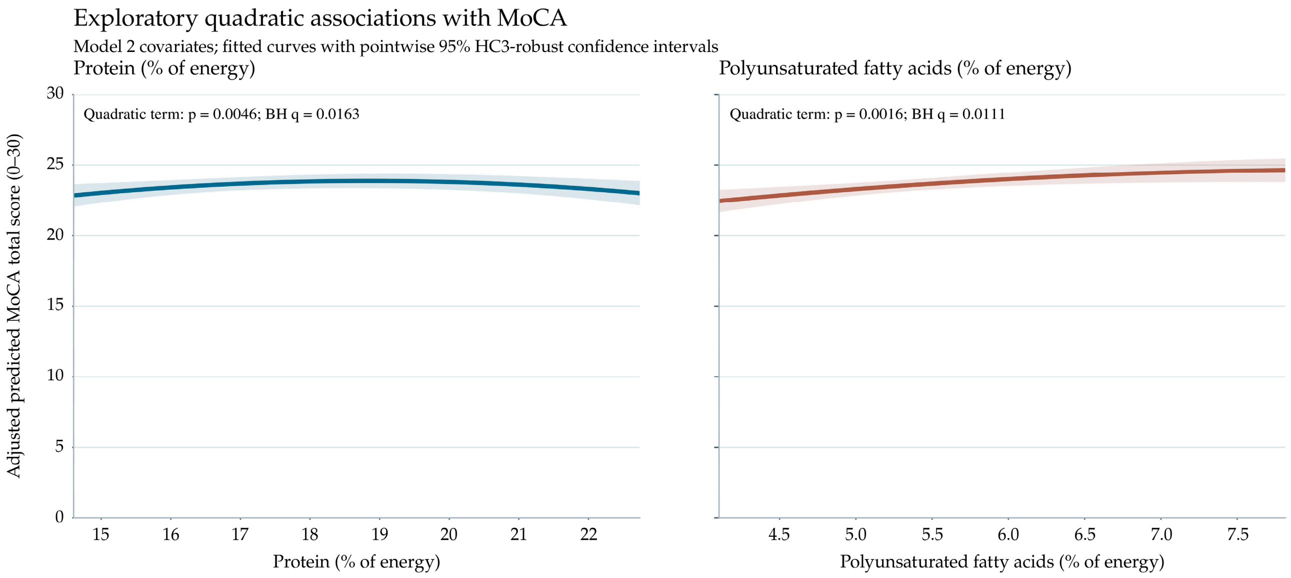

Model coefficients with HC3 robust 95% confidence intervals (n = 286)

Exposure	Linear B (95% CI)	Quadratic B (95% CI)	p	BH q
Protein (E%)	2.1231 (0.6477 to 3.5985)	−0.0563 (−0.0952 to −0.0175)	0.0046	0.0163
PUFA (E%)	2.0317 (0.8262 to 3.2372)	−0.1214 (−0.1964 to −0.0465)	0.0016	0.0111

Separate models adjusted for age, sex, and total energy intake; both nutrient terms were included together.
Exposures were not centered. Linear B: MoCA points per E%; quadratic B: MoCA points per $(E\%)^2$.
Coefficient CIs and quadratic-term p values use t inference (280 df); BH q values adjust seven quadratic-term tests.
Curves span the 5th–95th exposure percentiles; shaded bands show pointwise approximate 95% confidence intervals.
E%, percentage of total energy; PUFA, polyunsaturated fatty acids; CI, confidence interval; BH, Benjamini–Hochberg.

Figure S3. Exploratory quadratic associations of protein and polyunsaturated fatty acid intake with cognitive performance. Lines show predicted MoCA total scores from separate models including linear and quadratic terms for each exposure, adjusted for age, sex, and total energy intake (n = 286). Shaded bands indicate pointwise 95% confidence intervals calculated using HC3 robust standard errors. Both exposures are expressed as percentages of total energy intake; curves span the 5th–95th percentiles of each exposure. Quadratic-term p and q values were 0.0046 and 0.0163 for protein and 0.0016 and 0.0111 for polyunsaturated fatty acids, respectively. These values match Table S7 and the annotations within the figure. The Benjamini–Hochberg procedure was applied across the seven quadratic terms evaluated. These sensitivity analyses are exploratory and are presented separately from the main models with linear exposure terms. Abbreviations: BH, Benjamini–Hochberg; HC3, heteroscedasticity-consistent covariance estimator, type 3; MoCA, Montreal Cognitive Assessment.

Table S7. Exploratory quadratic associations between dietary composition and MoCA.

Dietary exposure and unit	Linear B (HC3 95% CI)	Quadratic B (HC3 95% CI)	Quadratic p value	BH-adjusted q value
Protein (% of total energy)	2.12312 (0.64772 to 3.59853)	−0.05633 (−0.09519 to −0.01747)	0.0046	0.0163
Carbohydrates (% of total energy)	−0.12404 (−0.71749 to 0.46942)	0.00055 (−0.00749 to 0.00858)	0.8935	0.8935
Total fat (% of total energy)	−0.01659 (−1.00422 to 0.97104)	0.00112 (−0.01029 to 0.01254)	0.8466	0.8935
Saturated fatty acids (% of total energy)	−1.22222 (−2.75195 to 0.30750)	0.04656 (−0.01038 to 0.10350)	0.1086	0.2535
Monounsaturated fatty acids (% of total energy)	0.30481 (−1.23359 to 1.84322)	−0.00464 (−0.04230 to 0.03302)	0.8086	0.8935
Polyunsaturated fatty acids (% of total energy)	2.03172 (0.82624 to 3.23720)	−0.12141 (−0.19636 to −0.04645)	0.0016	0.0111
Dietary fiber (g/1000 kcal)	−0.39954 (−1.26202 to 0.46294)	0.01101 (−0.02115 to 0.04317)	0.5009	0.8765

Linear and quadratic coefficients from seven separate models adjusted for age, sex, and total energy intake. All models included 286 participants. Each model included the exposure and its squared term simultaneously, with adjustment for age, sex, and total energy intake. Exposures were not centered. Protein, carbohydrates, total fat, and fatty acids were entered as percentages on a 0–100 scale; dietary fiber was entered as g/1000 kcal. B denotes the unstandardized regression coefficient. Units are MoCA points per percentage point for the linear term and per squared percentage point for the quadratic term; for fiber, the corresponding units are MoCA points per g/1000 kcal and per $(g/1000\ kcal)^2$. Linear and quadratic terms should be interpreted jointly. Confidence intervals and two-sided p values were calculated using HC3 heteroscedasticity-consistent standard errors and a t distribution with 280 residual degrees of freedom. Benjamini–Hochberg correction was applied to the seven quadratic-term p values using their unrounded values. The p and q columns refer exclusively to the quadratic terms. These analyses are exploratory. Abbreviations: B, unstandardized regression coefficient; BH, Benjamini–Hochberg; CI, confidence interval; HC3, heteroscedasticity-consistent covariance estimator, type 3; MoCA, Montreal Cognitive Assessment.

Table S8. Sensitivity analyses of associations between absolute fat intake and FRAIL categories, additionally adjusted for total energy intake.

Dietary exposure (g/day)	N	Adjusted OR	95% CI	p-value	Parallel lines test, p
Total fat	290	0.9787	0.9580–0.9998	0.048	0.943
Saturated fatty acids (SFA)	290	0.9575	0.9104–1.0069	0.091	0.926
Monounsaturated fatty acids (MUFA)	290	0.9748	0.9395–1.0116	0.177	0.849
Polyunsaturated fatty acids (PUFA)	290	0.9653	0.9004–1.0349	0.321	0.869

Separate proportional odds ordinal logistic regression models were fitted for FRAIL categories (0, 1, 2, and ≥3), adjusting for age, sex, and total energy intake. Each dietary exposure was evaluated separately. Odds ratios represent a 1-g/day higher intake; OR <1 indicates lower odds of being in a higher FRAIL category. Two-sided Wald p-values are reported without adjustment for multiple testing; these sensitivity analyses were exploratory. Parallel lines tests showed no evidence of violation of the proportional odds assumption.

Abbreviations: CI, confidence interval; MUFA, monounsaturated fatty acids; OR, odds ratio; PUFA, polyunsaturated fatty acids; SFA, saturated fatty acids.
